# Limits on inferring T cell specificity from partial information

**DOI:** 10.1073/pnas.2408696121

**Published:** 2024-10-07

**Authors:** James Henderson, Yuta Nagano, Martina Milighetti, Andreas Tiffeau-Mayer

**Affiliations:** ^a^Division of Infection and Immunity, University College London, London WC1E 6BT, United Kingdom; ^b^Institute for the Physics of Living Systems, University College London, London WC1E 6BT, United Kingdom; ^c^Division of Medicine, University College London, London WC1E 6BT, United Kingdom; ^d^Cancer Institute, University College London, London WC1E 6DD, United Kingdom

**Keywords:** TCR, immune repertoire, information theory, Renyi information, receptor-ligand interaction

## Abstract

A key challenge in molecular biology is to decipher the mapping of protein sequence to function. To perform this mapping requires the identification of sequence features most informative about function. Here, we quantify the amount of information (in bits) that T cell receptor (TCR) sequence features provide about antigen specificity. We identify informative features by their degree of conservation among antigen-specific receptors relative to null expectations. We find that TCR specificity synergistically depends on the hypervariable regions of both receptor chains, with a degree of synergy that strongly depends on the ligand. Using a coincidence-based approach to measuring information enables us to directly bound the accuracy with which TCR specificity can be predicted from partial matches to reference sequences. We anticipate that our statistical framework will be of use for developing machine learning models for TCR specificity prediction and for optimizing TCRs for cell therapies. The proposed coincidence-based information measures might find further applications in bounding the performance of pairwise classifiers in other fields.

## Significance

The specificity of cellular immune responses is determined by the binding of T cell receptors (TCRs) to diverse ligands, yet due to their vast diversity, most TCRs lack experimentally validated binding partners. To overcome this gap requires understanding the recognition code linking receptors and ligands. Here, we introduce an information theoretic approach to rank TCR features by their relevance to predicting specificity and bound how accurately T cell specificity can be predicted from partial information. By identifying informative features, our work provides a rational basis for prioritizing matches in TCR databases and for developing machine learning models to predict TCR–ligand interactions.

Mapping the amino acid sequence of a particular T cell receptor (TCR) to its antigen specificity is a holy grail of systems immunology (1–3). The T cell receptor endows T cells with the ability recognize snippets of pathogenic material presented on the surface of antigen-presenting cells by major histocompatibility complexes (MHC) (4). TCRs are specific, meaning a given T cell will only activate in response to a select range of antigen stimuli. Coverage of the vast antigen space explored by evolving pathogens is enabled by immense sequence variation within the TCR (5, 6), in particular within six hypervariable loops of the heterodimeric receptor, named complementarity determining regions (CDRs).

The immense diversity of TCRs implies that many have no experimentally determined ligands (7). Emerging computational approaches predict the specificity of such orphan TCRs by their sequence similarity to annotated TCRs (1, 3, 8, 9). However, which level of partial matching is sufficient for reliable prediction has remained unclear. Moreover, there is substantial interest in understanding for which immunological questions knowledge of paired receptor chains obtainable by single-cell sequencing is worth the trade-off with the higher throughput achievable by bulk sequencing (10) and which TCR features are most informative for machine learning applications (3, 11).

**Fig. 1.**
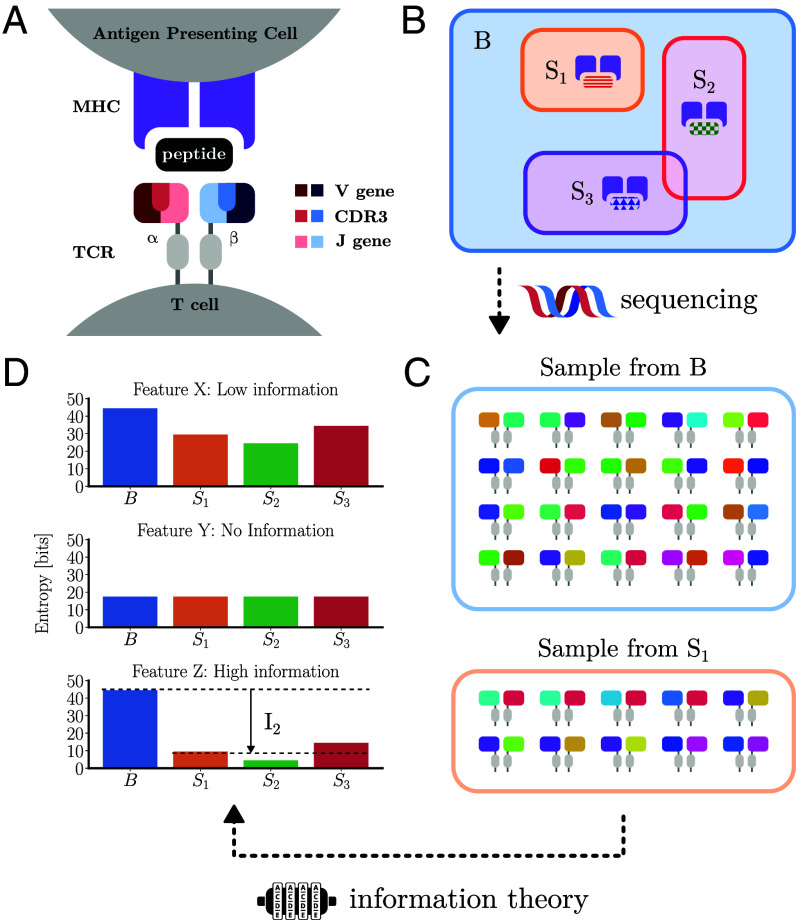
Overview of analysis methodology. (*A*) Sketch of T cell receptor structure highlighting the V, CDR3, and J regions and their interaction with MHC-bound peptides. The TCR is composed of two chains, most commonly α and β chains. Each chain is generated by the process of V(D)J recombination during T cell development, which combines a V (variable), J (joining), and C (constant) gene, with the addition of a D (diversity) gene in the β chain. Within each chain, the CDR1 and CDR2 amino acid loops are coded for by the V gene while the CDR3 regions are at the V(D)J intersection, which is additionally diversified through the random insertion and deletion of nucleotides at gene template junctions. (*B*) An abstracted view of TCR sequence space. The set B includes all possible TCRs. The subsets S_*i*_ represent TCRs specific to particular ligands. (*C*) Sequencing TCR from either the whole repertoire or epitope-specific subsets gives us samples from their respective distributions. (*D*) The number of pairs which match in a particular feature may then be recorded to compute a probability of coincidence. The logarithm of the probability of coincidence gives a measure of the entropy of the feature. Our information theoretic approach quantifies the change in entropy between background TCRs and sets of specific TCRs of different features (*Top* to *Bottom*). Features which experience a large reduction in entropy (*Bottom*) are the most informative for predicting the epitope specificity of a sequence.

Here, we address these important open questions by putting universal limits on the accuracy with which TCR specificity can be predicted from partial information. Our work takes inspiration from a long history of successful applications of information theory to the study of complex biological input–output relationships from neural coding (12–14) and transcriptional regulation (15, 16) to pattern formation during embryo development (17–19). Following recent applications of information theory to TCR repertoires by us (20) and others (21), our analysis builds on a fundamental insight from evolutionary biology: Patterns of sequence conservation in protein families provide clues about functionally relevant properties. In the immunological context, this means that TCR features that are important for specific recognition of a particular epitope will often be highly conserved among epitope-specific TCRs relative to their global diversity (Fig. 1).

In our current work, we provide the first comprehensive map of how much information each section of the paired chain TCR sequence provides about its specificity. To provide such a map, we make use of two recent datasets that have sequenced TCRs specific to a dozen viral MHC class I epitopes (1, 22). We overcame statistical limitations of prior analyses to pairs of residues (20, 21, 23) using coincidence-based measures of repertoire diversity (24). These measures can be estimated from smaller samples than traditional measures based on Shannon entropy (25–27). The information-theoretic approach naturally allowed us to identify synergies between different TCR sections in determining antigen specificity. Importantly, our quantification of coincidence information is underpinned by theory that directly links achievable classification accuracy to the coincidence information gained from a partial match and prior beliefs about the prevalence of epitope-specific T cells in a repertoire.

## 1. An Information-Theoretic Approach to T Cell Specificity

### 1.1. Coincidence Analysis for Features.

We have recently introduced a coincidence-based statistical framework to measure antigen-driven selection in TCR repertoires (24). The main idea of this work was to quantify clonal convergence by counting how often pairs of independently recombined clonal lineages in a sample have TCRs that are more similar to each other than some threshold level. Here, we pursue a conceptually related but unique approach that considers near-coincidences as coincidences on the level of coarse-grained TCR features. A feature may be a gene segment choice at a given locus, an amino acid at a particular residue, or a physical property of a hypervariable loop such as its charge or length. Features may also contain other features such as the α chain containing the Vα, Jα, and the CDR3α as component features.

Mathematically, a feature is a random variable that maps the sample space of all TCR sequences to a discrete set of possible categories. We denote the measure on the feature set for randomly drawn TCRs from a repertoire by P(X). The probability that two independent draws return the same outcome, i.e., the probability of coincidence of X, is then defined by

[1] $$\begin{array}{c} p_{C}\left[X\right]=\sum_{x}P{\left(x\right)}^{2}, \end{array}$$

where P(x) represents P(X=x) and the sum runs over all possible outcomes of X. We recall that in ecology, $p_{C}\left[X\right]$ is referred to as the Simpson’s diversity index of X with $D_{2}\left[X\right]=1/p_{C}\left[X\right]$ being an effective number of distinct species in a population (28).

Intuitively, we expect the most informative features to be those whose diversity is most reduced among TCRs specific to the same epitope when compared with background TCRs. In the following, we will make this intuition mathematically precise using a coincidence-based formulation of information theory.

We note that feature importance in this information-theoretic sense is not necessarily synonymous with the biophysical importance of a feature for the receptor–ligand interaction, as there are often multiple binding solutions for a given ligand (1, 24): TCR properties involved in binding but variable across solutions might not be globally informative in the way considered here. We will revisit the impact of the multiplicity of binding solutions on our information-theoretic measures in Section 3.4.

### 1.2. Coincidence Entropy.

A central quantity in information theory is entropy. The entropy of a probability distribution P(X) is given in its form proposed by Shannon in 1948 as (29)

[2] $$\begin{array}{c} H\left[X\right]=\sum_{x}P\left(x\right)\log P\left(x\right). \end{array}$$

Entropy represents the average amount of information lacking about the outcome of a measurement of discrete random variable X. It is usually calculated with the logarithm taken to base 2 such that its units are in bits and all logarithms in the following should be understood as logarithms taken with respect to this base. In 1961, Renyi showed that by relaxing one of the Shannon–Khinchin axioms from which the mathematical form of entropy is uniquely derived (strong additivity), a more general expression for entropy may be obtained (30, 31)

[3] $$\begin{array}{c} H_{\alpha }\left[X\right]=\frac{1}{1-\alpha }\log\left(\sum_{x}P{\left(x\right)}^{\alpha }\right), \end{array}$$

where α is referred to as the order of the Renyi entropy. The family of Renyi entropies include Shannon’s entropy measure as the limit of α→1.

We may note that the probability of coincidence introduced in the previous subsection provides a measure for the Renyi entropy of order α=2

[4] $$\begin{array}{c} H_{2}\left[X\right]=-\log p_{C}\left[X\right]. \end{array}$$

The Renyi entropy of order 2 is known as collision entropy in cryptography and may also be motivated from an optimal code length perspective with nonlinearly weighted length penalties (32). Here, we use the term coincidence entropy to stress its relation to coincidence-counting among sample pairs. We focus on this entropy measure in the following as we will show that it relates directly to pairwise classification. For a generalization to higher-order Renyi entropies, see *SI Appendix*, Text 4.

### 1.3. Coincidence Mutual Information.

We have previously used the coincidence ratio $p_{C}\left[X|\Pi \right]/p_{C}\left[X\right]$ between specific and background TCRs as a measure of antigen-driven selection (24), where $p_{C}\left[X|\Pi \right]$ is the probability of coincidence among epitope-specific TCRs averaged over a distribution of epitopes, P(Π), and $p_{C}\left[X\right]$ the probability of coincidence among background TCRs. Different definitions of conditional Renyi entropy for α≠1 have been proposed. Here, we follow refs. 33 and 34 and define

[5] $$\begin{array}{c} H_{2}\left[X|Y\right]=-\log p_{C}\left[X|Y\right], \end{array}$$

where $p_{C}\left[X|Y\right]$ is an average of $p_{C}\left[X|y\right]$ over all outcomes y of Y

[6] $$\begin{array}{c} p_{C}\left[X|Y\right]=\sum_{y}\rho_{2}\left(y\right)p_{C}\left[X|y\right], \end{array}$$

with weighting factors

[7] $$\begin{array}{c} \rho_{2}\left(y\right)=\frac{P{\left(y\right)}^{2}}{\sum_{y}P{\left(y\right)}^{2}}. \end{array}$$

Detailed justification for these definitions is provided in *SI Appendix*, Text 1. This definition allows us to express the coincidence probability ratio in terms of coincidence entropies

[8] $$\begin{array}{c} \log\left(\frac{p_{C}\left[X|\Pi \right]}{p_{C}\left[X\right]}\right)=H_{2}\left[X\right]-H_{2}\left[X|\Pi \right]. \end{array}$$

We note that for Shannon entropy this difference defines the mutual information between X and Π (29), which motivates the following definition of *coincidence mutual information*

[9] $$\begin{array}{c} I_{2}\left(X,\Pi \right)=\log\left(\frac{p_{C}\left[X|\Pi \right]}{p_{C}\left[X\right]}\right). \end{array}$$

Importantly, our definition of conditional entropy maintains additivity $H_{2}\left[X,Y\right]=H_{2}\left[X\right]+H_{2}\left[Y|X\right]$, where $H_{2}\left[X,Y\right]$ is the coincidence entropy of P(X,Y), the joint distribution of the random variables X and Y. As a correlate it follows that coincidence mutual information is symmetric, $I_{2}\left(X,Y\right)=I_{2}\left(Y,X\right)$—as is its Shannon counterpart—so it tells us not only how much information we gain about sequence features upon learning their epitope specificity, but also, by symmetry, how much information a sequence feature provides about its epitope specificity. Coincidence mutual information thus provides a natural way to score the importance of a TCR feature in predicting specificity, which we will refer to as the feature relevancy.

### 1.4. Describing the Interactions between Features with Redundancy and Synergy.

The connection between coincidence analysis and information theory naturally allows us to apply additional notions from information theory (35, 36) to describe how multiple features work in tandem to provide antigen specificity. First, conditional mutual information

[10] $$\begin{array}{c} I_{2}\left(X,\Pi |Y\right)=H_{2}\left[X|Y\right]-H_{2}\left[X|\Pi ,Y\right], \end{array}$$

describes the remaining information provided by feature X given that the value of a second feature Y is already known. Here, $H_{2}\left[X|\Pi ,Y\right]$ indicates conditioning on both epitope specificity and feature Y. If $I_{2}\left(X,\Pi |Y\right)=0$, then we refer to X as a fully redundant feature in the context of Y. As a trivial example, knowledge of the complete primary sequence of the full α chain makes any information provided by CDR3α redundant, and so on.

Second, interaction information

[11] $$\begin{array}{c} I_{2,\mathrm{int}}\left(X,Y|\Pi \right)=I_{2}\left([X,Y],\Pi \right)-I_{2}\left(X,\Pi \right)-I_{2}\left(Y,\Pi \right) \end{array}$$

describes how much additional information both features provide in conjunction (*SI Appendix*, Text 2). Here, $I_{2}\left([X,Y],\Pi \right)$ is the relevancy of the feature produced by combining the two features X and Y. If $I_{2,\mathrm{int}}\left(X,Y|\Pi \right)>0$, then there is synergy between the two features.

## 2. Bounding Classification Accuracy of Partial TCR Matches

### 2.1. Pairwise Classification Odds.

There are well-known connections between information measures and achievable classification errors both in the Shannon (37) and Renyi case (38, 39). In the following, we derive how TCR classification accuracy using partial feature matches with a reference sequence is bounded when only partial information is available. We consider a classification setting where the task is to identify spiked-in TCR sequences specific to a particular epitope π in an otherwise naive repertoire. We will derive how posterior classification odds depend on feature relevancy and prior beliefs, i.e., the fraction of spiked-in sequences P(π). Mathematically, in this setting, the presence of a TCR sequence σ is due to either of two generative processes:

[12] $$\begin{array}{c} P(\sigma )=P(\pi )P(\sigma |\pi )+P(B)P(\sigma |B), \end{array}$$

where P(B)=(1−P(π)) and where P(σ|π) is the probability of drawing σ from the distribution of TCR sequences specific to epitope π, P(Σ|π), and P(σ|B) the probability of drawing σ from the distribution of background TCR sequences according to V(D)J recombination, P(Σ|B). In practice, we used a computational model to determine the probability of generation of TCR sequences P(Σ|B) (40). This choice of background yields a distribution without the imprints of thymic or peripheral selection that determine TCR coincidence probabilities in naive and memory repertoires (24).

To recapitulate the empirical procedure of matching TCR sequences to a database of known binders, we consider the following one-shot classification strategy: We classify a query sequence as having been generated from P(Σ|π), if it matches in a feature X with a reference sequence randomly drawn from P(Σ|π). Using the odds formulation of Bayes’ theorem, we may express the posterior odds of correct classification as

[13] $$\begin{array}{c} \frac{P(\pi |x=x^{\prime })}{P(B|x=x^{\prime })}=\frac{P(x=x^{\prime }|\pi )}{P(x=x^{\prime }|B)}\frac{P(\pi )}{P(B)}. \end{array}$$

Here, $P\left(x=x^{\prime }|\pi \right)=p_{C}\left[X|\pi \right]$ is the probability of a match in feature X if both sequences were truly drawn from distribution P(Σ|π), while $P(x=x^{\prime }|B)$ is the probability of a match in feature X for a query drawn from P(Σ|B) and a reference drawn from P(Σ|π). Under the assumption that the propensity of a TCR for specific binding is independent of its recombination probability (24), one can show that $P\left(x=x^{\prime }|B\right)=p_{C}\left[X\right]$ (*SI Appendix*, Text 3.A). Therefore,

[14] $$\begin{array}{c} \frac{P(\pi |x=x^{\prime })}{P(B|x=x^{\prime })}=\frac{p_{C}\left[X|\pi \right]}{p_{C}\left[X\right]}\frac{P(\pi )}{P(B)} \end{array}$$

This expression can be generalized for mixtures of multiple epitope groups, in which case the average odds over epitopes (*SI Appendix*, Text 3.B) can be expressed as

[15] $$\begin{array}{c} \langle \frac{P(\pi |x=x^{\prime })}{P(B|x=x^{\prime })}\rangle =\frac{p_{C}\left[X|\Pi \right]}{p_{C}\left[X\right]}\langle \frac{P(\pi )}{P(B)}\rangle , \end{array}$$

where $p_{C}\left[X|\Pi \right]$ is the conditional probability of coincidence defined previously and the averages for the odds are taken over P(π)/(1−P(B)). By the definition of coincidence mutual information (Eq. **9**), we can rewrite the last equation as

[16] $$\begin{array}{c} O_{\;\text{post}}=2^{I_{2}\left(X,\Pi \right)}\;O_{\;\text{prior}}, \end{array}$$

which links the average posterior odds $O_{\;\text{post}}$ to average prior odds $O_{\;\text{prior}}$ via coincidence mutual information. Each bit of coincidence mutual information between X and Π corresponds to a two-fold gain in posterior odds.

### 2.2. When Is Partial Information Sufficient?.

Eq. **16** captures an important Bayesian intuition about classification: Correct classification depends not only on how much information we have available but also on our prior belief. Here, our prior belief about the likelihood that any particular sequence is specific should reflect the total fraction of spiked-in sequences. If we are searching for a needle in a haystack, this is when $O_{\mathrm{prior}}$ is small, we need to use more highly informative features for correct classification. Mathematically, a minimal prior odds of $2^{-I_{2}\left(X,\Pi \right)}\;T$ is needed to ensure that the average posterior odds exceeds a threshold value T. Expressed in terms of prior probabilities

[17] $$\begin{array}{c} P_{\;\text{prior}}\left(I_{2}\right)\geq \frac{T\;2^{-I_{2}}}{1+T\;2^{-I_{2}}}, \end{array}$$

is needed if only $I_{2}$ bits are available for classification. To illustrate this result, we performed in silico simulations with a toy model of TCR specificity (*SI Appendix*, Text 3.D). These simulations showed close agreement between predicted values for $P_{\mathrm{prior}}\left(I_{2}\right)$ and those obtained through numerical simulation (*SI Appendix*, Fig. S1).

Note that sequences drawn from P(Σ|B) may also be specific to π. Therefore, P(π) and $P(\pi |x=x^{\prime })$ are not exactly equal to the fraction of sequences specific to π and the posterior probability of specificity, respectively. However, as shown in *SI Appendix*, Text 3.C in most cases of practical interest, where P(π) exceeds the background frequency of sequences specific to a given epitope, this distinction is irrelevant.

## 3. Application of the Methodology to TCR Sequence Data

To illustrate how our framework can be applied, we curated a dataset of multimer-sorted TCRs from CD8^+^ T cells with specificity to viral antigens (*SI Appendix*, Text 7). We restricted our dataset to epitopes where at least one full TCR coincidence was observed to allow computation of coincidence information for the full TCR. Remarkably, such coincidences are observed in many epitope-specific repertoires: Here, we combined nine SARS-CoV-2-specific repertoires with such coincidences studied by Minervina et al. (22) with three repertoires specific to other viral epitopes from Dash et al. (1). To obtain background TCRs, we randomly paired TCRα and TCRβ sequences generated by a computational model of V(D)J recombination (40).

### 3.1. A Decomposition of TCR Specificity into Its Component Parts.

**Fig. 2.**
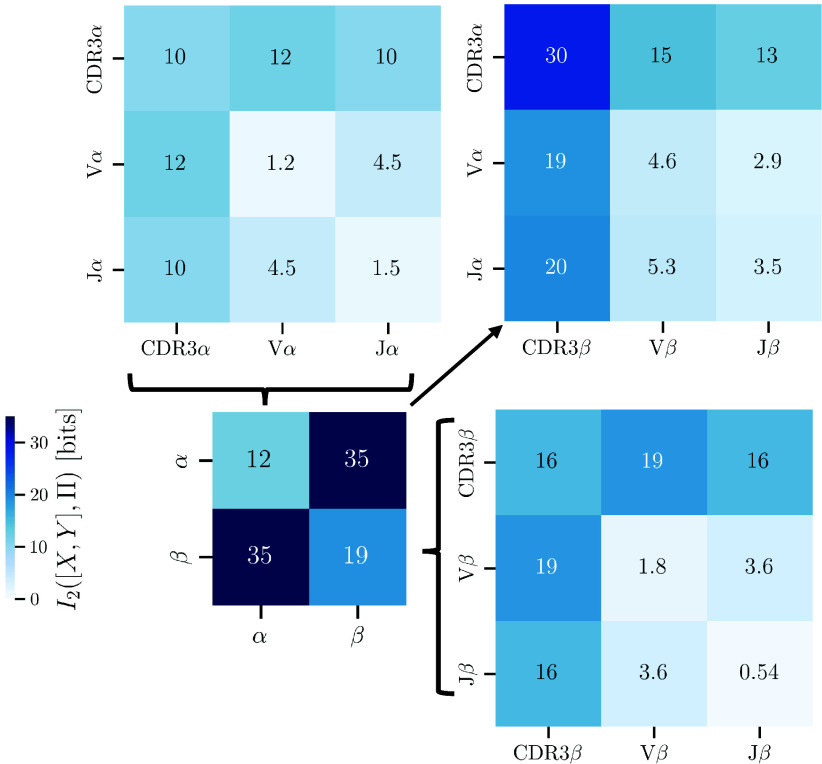
Coincidence mutual information between TCR sections and antigen specificity. Relevancy scores of various sections of the T cell receptor sequence. The off-diagonal values indicate the amount of coincidence information that combinations of features provide. The *Top Right* hand grid shows the relevancy of combination of features where one is from the α chain and the other the β chain. Interaction information between features can be computed by taking the difference between the off-diagonals and the sum of the corresponding diagonal values (Fig. 3).

To provide a top–down decomposition of the information content of the TCR, we computed the relevancy of different sections of the TCR for its specificity, as well as their combinations (Fig. 2). We first analyzed the information provided by the α and β chains alone which recapitulated the expected greater relevancy of the β chain (19 bits) than the α chain (12 bits). By Eq. **17**, the information provided by each chain bounds prior probabilities needed for accurate classification using single chain matches. A β chain match requires a prior probability $P_{\mathrm{prior}}\geq 3\cdot 10^{-5}$ for a 95% posterior confidence. In contrast, an α chain match allows reliable classification only for prior probabilities $P_{\mathrm{prior}}\geq 3\cdot 10^{-3}$. We then broke down the two chains further into their component V and J gene segments and CDR3 amino acid sequence. A CDR3β match provides 16 bits of information (corresponding to $P_{\mathrm{prior}}\geq 4\cdot 10^{-4}$) while a CDR3α match provides only 10 bits of information (corresponding to $P_{\mathrm{prior}}\geq 1\cdot 10^{-2}$).

In addition to features such as the CDR3, V, and J regions, our definition of a feature also extends to physical properties of the TCR such as the length of the CDR3 loop and its net charge (*SI Appendix*, Fig. S3). Our results confirm that these properties, which have been described in the literature as being important for epitope specificity (1, 41), have some relevancy in determining TCR specificity. For instance, CDR3β net charge is roughly as informative as Jβ choice. However, neither property captures a substantial proportion of CDR3 information demonstrating the contribution of higher-order sequence features to specific binding (*SI Appendix*, Text 6).

To assess variations in feature relevancy across epitopes, we defined local relevancy, $i_{2}\left(X,\pi \right)=\;\log\left(p_{C}\left[X|\pi \right]/p_{C}\left[X\right]\right)$, as the information gain for a specific epitope π. Local relevancy scores revealed a broadly consistent hierarchy of feature relevancy across epitopes (*SI Appendix*, Figs. S4–S8). The analysis also identified variability in local relevancy of features between different epitopes not explained by finite sampling deviations alone in line with our prior findings on a subset of the studied epitopes (27). We will analyze this variability in more detail in Section 3.4.

### 3.2. Synergy and Redundancy between TCR Features.

**Fig. 3.**
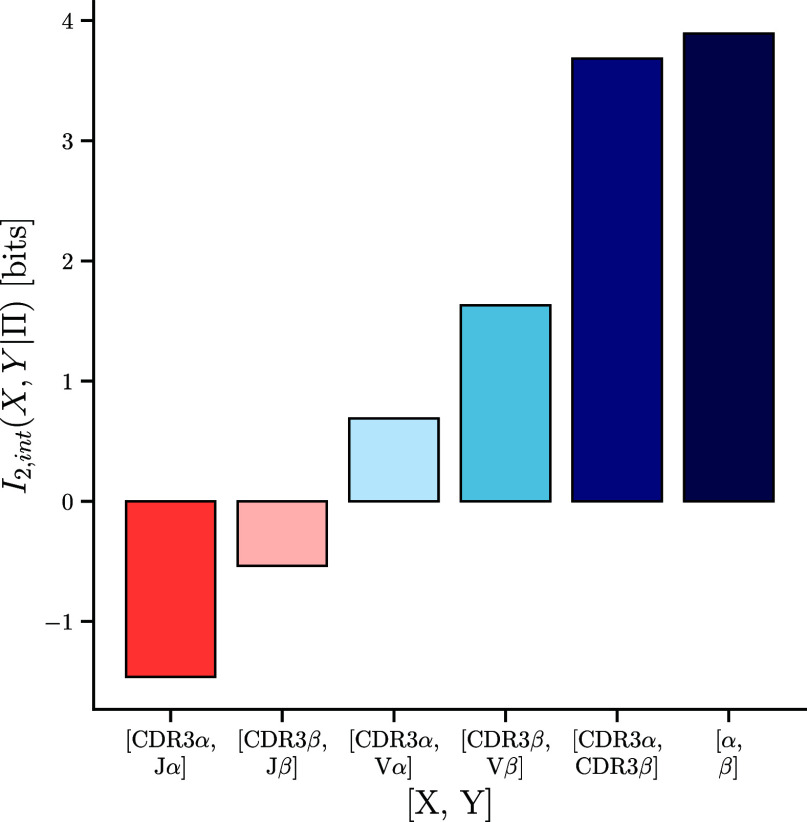
Synergistic and redundant TCR sequence features. Interaction information scores for combinations of features. Positive interaction information indicates that two features become more informative in the context of one another and hence have synergy while negative interaction information suggests redundancy between the features.

Comparing relevancy scores for individual and combined features revealed the pervasiveness of interactions between TCR sections (Fig. 2), where their combined information differed from the sum of their individual relevancies. Fig. 3 summarizes the interaction information between important TCR features.

Our analysis identified substantial synergy between the α and β chains (4 bits). This synergy implies that there are pairing restriction between α and β chains in specific TCRs, which make each chain more informative when considered in its full paired chain sequence context (*SI Appendix*, Text 2). These results broaden our prior findings (24) to a broader set of epitopes, and add to a growing literature investigating TCR α-β pairing rules (20, 24, 41–44). Pairing restrictions imply that the diversity of TCRs responding to a given epitope is lower than the product of the diversities of responsive α and β chains.

We also analyzed the interaction information between the CDR3 of each chain and the corresponding V segment choice. We again identified substantial synergy, presumably reflecting spatial constraints between V-gene encoded framework and CDR1/2 variability and CDR3 choice. In contrast, the interaction information between the CDR3 and J gene is negative. This is expected as the sequence variability provided by the J gene is contained in the CDR3 region (45) but demonstrates how our framework can identify redundant features without such a prior knowledge.

### 3.3. CDR3 Compression and Information Loss.

We next sought to determine how much information about specificity is lost when compressing the CDR3 amino acid sequence into reduced representations of different complexities. We took conceptual inspiration from the information bottleneck method, which posits a trade-off between compression and preserved information (46). As good compression schemes retain relevant features such analyses can provide insights into which properties of the CDR3 sequence matter for its specificity.

**Fig. 4.**
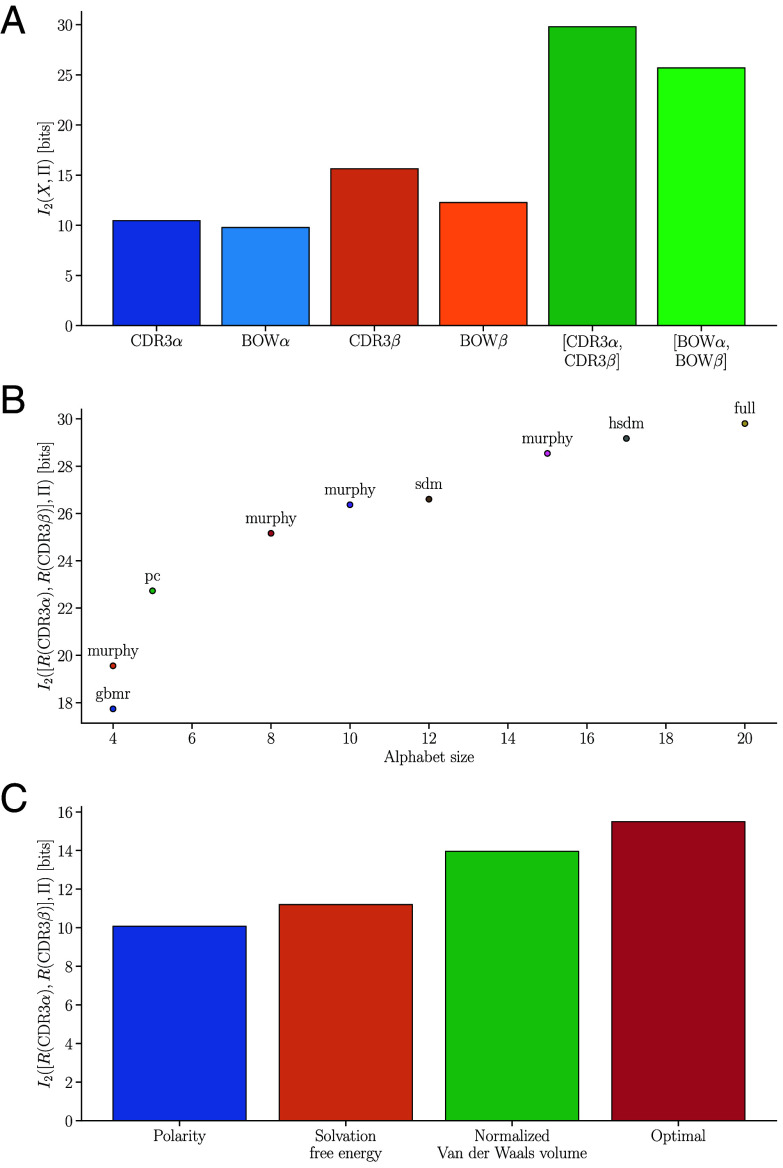
Information preserved by different CDR3 compression schemes. (*A*) Relevancy of bag-of-words (BOW) representations for the CDR3α, CDR3β, and both CDR3 chains. CDR3 bag-of-words representations are vectors of dimension 20 with each entry representing the number of occurrences of a particular amino acid. (*B*) Information retained when compressing CDR3s using reduced amino acid alphabets described in *SI Appendix*, Text 7.B. R(CDR3) denotes the CDR3 remapped to the reduced alphabet. (*C*) Information retained by different two letter alphabets for both CDR3 chains. Polarity, solvation free energy, and normalized Van der Waals volume alphabets are obtained by hierarchical clustering of amino acids with respect to these biophysical properties. (For further properties and different alphabet sizes, see *SI Appendix*, Table S3.) The optimal alphabet is obtained by a greedy search algorithm described in *SI Appendix*, Text 7.D.

First, we removed positional information by representing the CDR3 sequence as an unordered collection of its individual amino acids, referred to as bag-of-words representations in natural language processing (47). Such compression loses 4 bits of information for the paired chain receptor (Fig. 4*A*), highlighting the importance of the ordering of amino acids within the TCR. We next determined the relevancy of single dimensions of the bag-of-word vector, this is the number of times individual amino acids occur in the sequence. TCR amino acid contents provided less than a single bit of information about specificity, with arginine, proline, and glycine being most informative when considering both chains (*SI Appendix*, Fig. S9). Interestingly, glycine and proline content have been previously described as important for determining TCR specificity (41, 48), and both are determinants of protein flexibility

Second, we compared different reduced amino acid alphabets (49–53), which map amino acids to a smaller number of groups (Fig. 4*B*). By quantifying the compression-information trade-off of different reduced alphabets, our results can help guide the choice of reduced alphabets for TCR applications, for instance by identifying Pareto optimal alphabets at a given alphabet size. We next determined how much information is preserved by reduced alphabets obtained via hierarchical clustering with respect to single biophysical properties (54, 55). Our analyses (described in detail in *SI Appendix*, Text 7.3) revealed major differences between the informativeness of such alphabets (Fig. 4*D* and *SI Appendix*, Table S3). For instance, steric properties such as radius of gyration of side chain or accessible surface area in a tripeptide resulted in particularly informative reduced alphabets across alphabet sizes. Finally, we implemented a greedy search algorithm to find the best two-letter alphabet (*SI Appendix*, Text 7.4). This algorithm identified a maximally informative two letter alphabet, which retains 15.5 bits of information (Fig. 4*D*), providing proof-of-concept for data-driven identification of an optimal coarse-graining strategy.

### 3.4. Variability in Interaction Information across Epitopes Is Explained by Mixture Models.

**Fig. 5.**
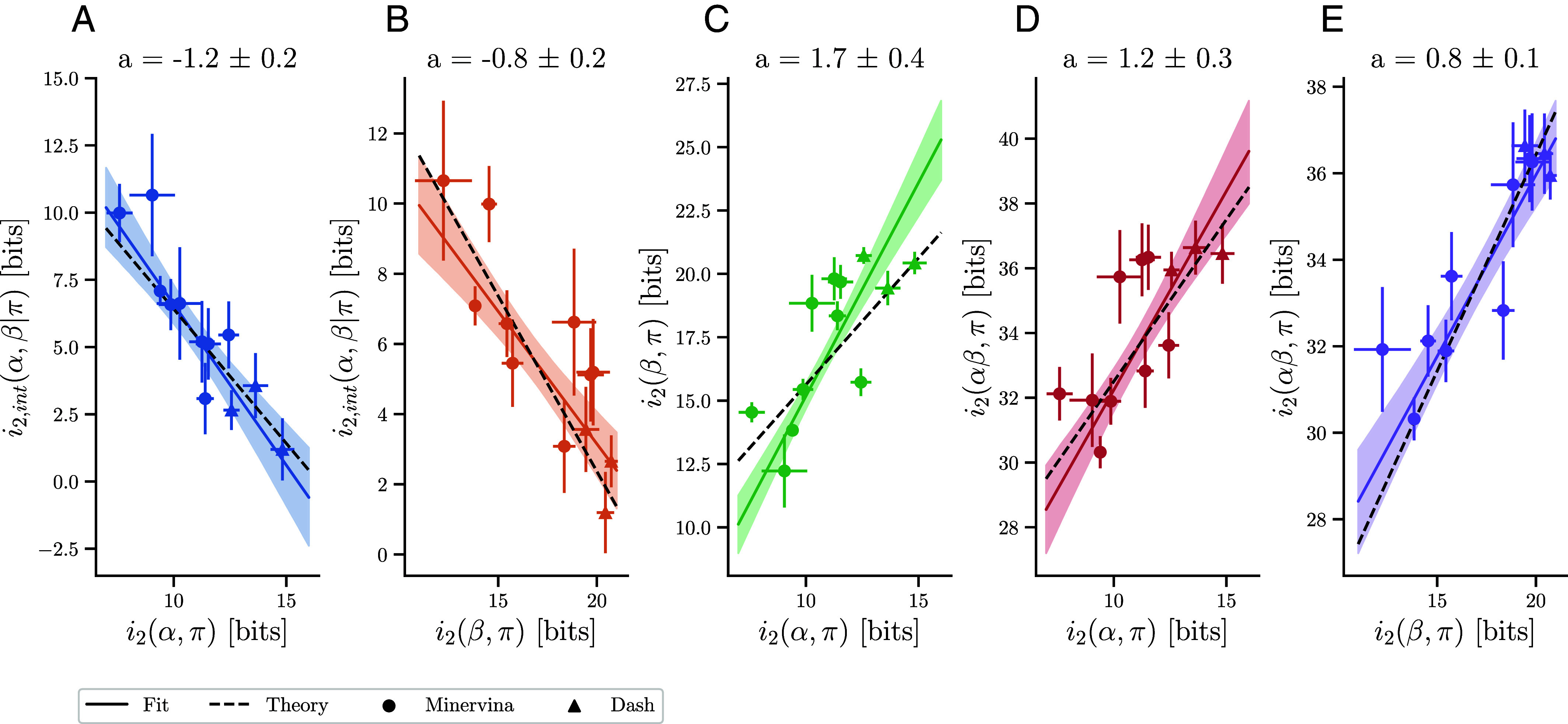
Correlation between α-β interaction information and per-chain information across epitopes. Local interaction information and single-chain information across epitopes. Weighted linear fits (solid lines) obtained using orthogonal distance regression were used to quantify the dependence between variables, with regression slopes a displayed above each panel. Epitope-specific interaction information depends negatively on the local informational value of the (*A*) α chain and (*B*) β. We furthermore find that the (*C*) per-chain relevancies are positively correlated with each other as is (*D* and *E*) total information with both single chain relevancies. The observed dependencies between variables agree well with theoretical expectations from a mixture model (dashed lines), in which epitopes differ in the number of distinct binding solutions or contain false positives.

To better understand potential sources of variability of TCR sequence restriction across epitopes we defined additional measures of local sequence variation: Local conditional mutual information $i_{2}\left(X,\pi |Y\right)=H_{2}\left[X|Y\right]-H_{2}\left[X|\pi ,Y\right]$ and local interaction information $i_{2,\;\text{int}}\left(X,Y|\pi \right)=i_{2}\left([X,Y],\pi \right)-i_{2}\left(X,\pi \right)-i_{2}\left(Y,\pi \right)$. We then analyzed dependencies across four variables (Fig. 5): Interaction information $i_{2,\;\text{int}}\left(\alpha ,\beta |\pi \right)$, α-chain relevancy $i_{2}\left(\alpha ,\pi \right)$, β-chain relevancy $i_{2}\left(\beta ,\pi \right)$ and paired chain relevancy $i_{2}\left([\alpha ,\beta ],\pi \right)$. These analyses highlighted strong dependencies between the variables. The more informative an α chain or β chain is for a given epitope, the less α-β interaction information contributes to global diversity restriction (Fig. 5 *A* and *B*). Moreover, epitopes with more informative α chains also have more informative β chains (Fig. 5*C*) and more informative full TCR sequences (Fig. 5*D*).

Unexpectedly, all variables were highly correlated with each other and well fitted by linear regressions, suggesting the existence of a single underlying degree of freedom that drives the observed variability across epitopes. Based on the clustering of epitope-specific TCRs, we had previously proposed mixture of motif models (24), in which epitope-specific TCRs are composed of a number of distinct binding solutions (binding modes or motifs). We asked whether variability in the number of such motifs across epitopes might provide the common degree of freedom explaining the observed correlations. Deriving the expected theoretical relationships between variables (*SI Appendix*, Text 6), we found an increased local interaction information for epitopes with more binding modes and a decrease in individual feature relevance. Across all variable pairs studied in Fig. 5, the mixture model predicted linear relations with slopes of ±1, in good agreement with the best fit lines to the empirical data. Intuitively, if an epitope has multiple binding solutions, more α and β chains will be able to bind it, given the right complementary chain (thus lowering the information from each individual chain). At the same time, where many solutions exist a high degree of α–β pairing is expected as most α chains from one binding solution would not be valid with β chains from another solution (thus increasing the observed synergy between the two chains).

Another consequence of the existence of multiple binding solutions is a potential loss of relevance of features with variable restriction across TCR clusters, as we have discussed when introducing our information-theoretic definition of feature relevance. In analyzing the relevancy of CDR3 length and charges as features (*SI Appendix*, Text 5), we found evidence for this phenomenon: The relevancy of length and charge increases substantially when conditioning on V and J gene usage, which acts as a simple proxy for distinguishing TCR clusters (*SI Appendix*, Fig. S2). These results further support modeling epitope-specific repertoires as mixtures.

Given the low prevalence of epitope-specific TCRs in a repertoire, we additionally expect the dataset to be a mixture containing some false positive TCRs with no or low affinity to the epitope of interest even if sorting has high specificity. As we show in *SI Appendix*, Text 6 variations in the proportion of false positives across epitopes can also explain the observed dependencies among variables, with high interaction information for epitopes with many false positives. Both models share the common underlying insight that epitope-specific repertoires are mixtures rather than draws from a unimodal distribution—future research might elucidate the contributions of the different underlying mechanisms to the observed variability.

## 4. Distance Metrics and Near-Coincidence Entropy

### 4.1. Generalization of Coincidence Mutual Information to Fuzzy Matches.

As exact matches are rare for complex features, it is of interest to also quantify the information provided by fuzzy feature matches. As previously explored in ref. 24, we are not limited to computing the probability of exact coincidences between features but can also consider near-coincidences according to some distance metric. Given a feature X distributed according to P(X) and a distance metric $d(x,x^{\prime })$ between outcomes x and $x^{\prime }$, the probability that two draws from P(X) are at distance $d(x,x^{\prime })=\Delta$ can be defined as

[18] $$\begin{array}{c} p_{C}\left[X\right]\left(\Delta \right)=\sum_{x,x^{\prime }}P\left(x\right)P\left(x^{\prime }\right)\delta_{d(x,x^{\prime }),\Delta }, \end{array}$$

where $\delta_{d(x,x^{\prime }),\Delta }$ is the Kronecker delta. We use this measure to propose a near-coincidence entropy $H_{2}\left[X\right]\left(\Delta \right)=-\log p_{C}\left[X\right]\left(\Delta \right)$, and a near-coincidence conditional entropy $H_{2}\left[X|Y\right]\left(\Delta \right)=-\log p_{C}\left[X|Y\right]\left(\Delta \right)$, where $p_{C}\left[X|Y\right]\left(\Delta \right)$ once again is an average of $p_{C}\left[X|y\right]\left(\Delta \right)$ over outcomes of Y using the $\rho_{2}\left(y\right)$ weighting factor. We define a near-coincidence mutual information

[19] $$\begin{array}{c} I_{2}\left(\Delta_{X,X^{\prime }},Y\right)=H_{2}\left[X\right]\left(\Delta \right)-H_{2}\left[X|Y\right]\left(\Delta \right), \end{array}$$

where $\Delta_{X,X^{\prime }}$ denotes that this information is computed for near-coincidences in feature X at distance Δ. As this measure deals with pairs of instances of a random variable rather than single instances, this quantity cannot be defined straightforwardly for Shannon entropy but is motivated naturally when using coincidence entropy.

### 4.2. Pairwise Classification Using Fuzzy Matches.

To obtain an interpretation of near-coincidence entropy, we turn once again to pairwise classification. We consider the same classification procedure as previously but based on a fuzzy match where the sequence with unknown specificity is distance Δ from the sequence with known specificity such that $d(x,x^{\prime })=\Delta$. Similarly to our prior derivations, we find (*SI Appendix*, Text 3.E)

[20] $$\begin{array}{c} O_{\;\text{post}}=2^{I_{2}\left(\Delta_{X,X^{\prime }},\Pi \right)}\;O_{\;\text{prior}}. \end{array}$$

**Fig. 6.**
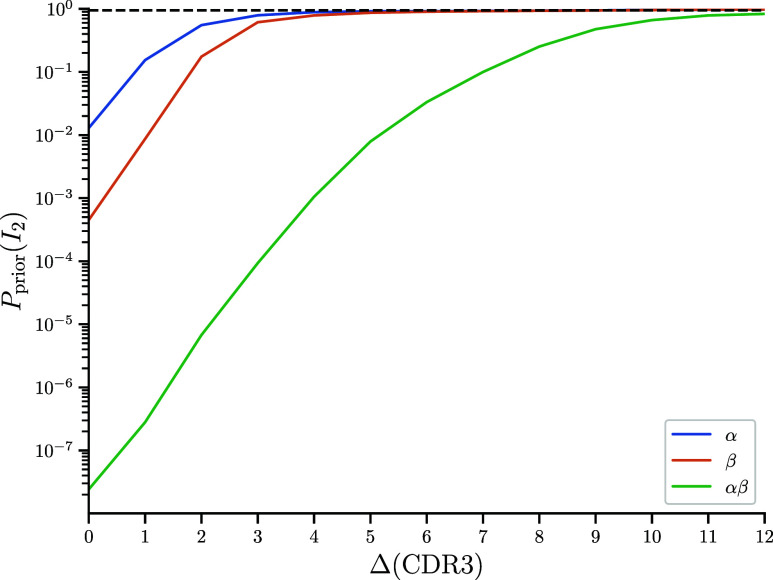
Information theoretic analysis of fuzzy CDR3 matches. Critical prior probabilities for 95% confidence in classification using a fuzzy match with a Levenshtein distance Δ. Distances are CDR3α and CDR3β amino acid edit distances as well as the sum of CDR3α+CDR3β edit distance. Levenshtein distances are defined as the minimum number of insertions/deletions and substitutions required to turn one sequence into another.

One bit of near-coincidence mutual information again corresponds to an average two-fold increase in posterior classification odds. As in the case of exact matching, Eq. **20** defines regimes in which fuzzy matches at a given distance are expected to succeed or fail. In Fig. 6, we provide an example of this by computing the required prior fraction of specific sequences to obtain a posterior probability of 0.95 with fuzzy CDR3 matches at a certain Levenshtein distance. Inversely, at a given prior odds ratio and target posterior odds ratio we can use these results to compute a critical distance beyond which classification becomes unreliable.

## 5. Discussion

The ubiquity of information theory lies in its ability to describe complex relationships between data points using a simple quantitative vocabulary. As shown by Shannon (29), entropy provides the most natural measure of uncertainty and hence changes in entropy directly capture how knowledge of one event increases understanding of another. The application of information theory to the problem of immune receptor specificity has proved highly fruitful in the past. In particular, estimates of residue Shannon entropy aided in identifying potential complementary determining regions of the TCR and immunoglobulin and highlighted that TCRs were the more diverse of these two antigen receptors (56). Other, more recent studies have employed concepts from information theory such as mutual information to quantify interactions between various sections of the TCR sequence (20, 21). These previous studies have taken a “bottom–up” approach, computing an upper bound on sequence diversity by summing up the entropy of each constituent amino acid residue or pairs of residues. In part, this “bottom–up” approach has been required due to biases in estimating Shannon entropy in small samples. Although there exist methods for reducing bias in Shannon entropy estimation, these still require resolving higher-order distribution moments or essentially resort to coincidence counting and use Renyi entropy to approximate Shannon’s (57, 58). In this work, we have proposed a “top–down” approach to decomposing TCR specificity firmly rooted in second-order Renyi entropy.

Our methodology provides a general framework to assess the role of individual TCR sequence features in determining antigen specificity as well as combinations of features by introducing the concepts of relevancy, redundancy, and synergy. We first compute the entropy of the full TCR sequence, divide this into its two constituent amino acid chains and then further subdivide these into their V, J, and CDR3 regions. Our results identify the β chain as the most informative of the heterodimeric TCR’s chains and the CDR3 regions to be the most informative regions of each chain. However, we also find that the information these constituent parts provide is far smaller than that of the full TCR sequence. Although these results are unsurprising, with previous work highlighting the higher contribution of the β chain in epitope binding predictions and the importance of paired chain data (23, 59, 60), we provide the first full quantification of the information contained within these regions and, as our methodology has its foundations in coincidence-based statistics, we are able to directly interpret information measures in terms of achievable pairwise classification accuracy. Our work thus paves the way for the development of principled Bayesian methods for interpreting partial sequence matches.

Our results provide clear guides for when a limited amount of TCR sequence information, such as a single chain, is on average enough to solve an epitope specificity classification problem and when this loss of information may seriously impact predictive performance. We expect these insights to be important for experimental design, to decide whether the time and cost trade-off of single cell sequencing over bulk are worth the increase in information paired chain information might provide.

We have also shown how the vocabulary of information theory can be applied to TCR near-coincidence analysis, which we have introduced in recent work (24). Our framework predicts pairwise classification performance when using fuzzy matches at a given threshold TCR distance. This approach may be used to define relevant data regimes in which current or future distance metrics (1, 61) may be usefully applied and allows setting critical distances for classifying or clustering sequences (62).

Our “top–down” approach allows us to compute interaction information, which describes synergistic and redundant relationships between TCR sequence features. We observe positive interaction information, synergy, between the α and β chain as well as the CDR3 and V regions, while knowledge of CDR3 regions makes their associated J regions redundant. We furthermore show how the relationship between interaction and single chain information across epitopes is compatible with a model in which epitopes vary in the number of distinct binding solutions (or possibly in the rate of false positives). With the steady accumulation of data on more epitopes, we envisage that our approach will help decipher principles underlying sequence space organization of responding TCRs.

Our framework can also be used to assess how much information is retained by compressed representations of the TCR such as bag-of-words vectors or reduced amino acid alphabets. We provide proof-of-concept for how our information scores may be used to construct reduced alphabets optimized for preserving epitope specific information and to discover biophysically salient measures of amino acid similarity.

The next steps for applying our theoretical approach are numerous. On the practical side, we propose completing the “top–down” approach and performing an analysis of the informational value of the CDR3 sequences residue by residue. This may allow for the identification of informationaly dense regions of the CDR3s and for a quantification of more complex allosteric interactions present across the receptor structure. Such analyses could complement work on structure-based prediction of TCR–pMHC interactions (63) and prediction with biophysical interaction energy models using contact maps derived from solved structures (64, 65). Further extensions of our framework could account for the hierarchy of selective processes shaping the TCR repertoire by varying the background used to compute background entropy. For example, to bound the performance of multiclass classification between a set of known epitopes, it may be more appropriate to quantify the entropy of TCRs across the chosen epitope-specific groups. Likewise, sequence statistics in a naive T cell repertoire could be used as background to account for the imprint of thymic selection. Our information theoretical tools may also be used on problems other than epitope specificity. For example, one may apply them to the study of TCR–MHC associations (66, 67) or TCR sequence to phenotype relationships (44, 68–70).

Linking feature information to classification isn’t a problem unique to the field of protein function nor is the task of class prediction from pairwise comparisons. Transformer neural networks, the architecture underlying the current rise of large language models, embed data in high dimensional vector spaces (71) and may be trained in a pairwise contrastive manner, such that items from the same class are closer together than items from different classes (72–75). More generally, metric and representation learning commonly utilize pairwise measures to tackle problems ranging from sentence embeddings to facial recognition (76–80). Our pairwise coincidence information measure may be applicable for identifying interpretable informative features in these applications.

To conclude, we have introduced a theoretical framework for mapping the information content of the T cell receptor sequence with regard to its antigen specificity. Our results confirm prior insights from more limited structural studies regarding the relative importance of the α and β chains (2, 5, 81–84) but also highlight unexpected variability in the synergy between chains across epitopes. As dataset sizes continue to increase, the proposed framework should be able to guide the training of protein language models for predicting TCR–pMHC specificity (85–87) by information-content-driven masking strategies and will provide a tool to find interpretable physical features learned by such models.

## Supplementary Material

Appendix 01 (PDF)

## Data Availability

Detailed source code and pre-processed data necessary to reproducing all results reported in this manuscript are available online at https://doi.org/10.5281/zenodo.13760163 (88). Antigen-specific TCR sequences were obtained from Dash et al. (1) as deposited in VDJdb (89), and from the *SI Appendix* of Minervina et al. (2).
