# Supplementary material for: Limits on inferring T cell specificity from partial information
Source: Proc Natl Acad Sci U S A. 2024 Oct 7;121(42):e2408696121. doi: 10.1073/pnas.2408696121 (PMC11494314; doi:10.1073/pnas.2408696121)
Supplement: Supplementary file 1 — Appendix 01 (PDF) [file pnas.2408696121.sapp.pdf]

PNAS

Supporting Information for

Limits on Inferring T-cell Specificity from Partial Information

James Henderson, Yuta Nagano, Martina Milighetti, Andreas Tiffeau-Mayer

E-mail: andreas.mayer@ucl.ac.uk

This PDF file includes:

Supporting text
Figs. S1 to S9
Tables S1 to S3
SI References

Supporting Information Text

1. Defining conditional coincidence entropy

We follow Refs. (1, 2) and define conditional Renyi entropy as

$$H_\alpha[X|Y] = f^{-1}\left(\sum_y \rho_\alpha(y) f\left(H_\alpha[X|y]\right)\right), \quad [1]$$

where $\rho_\alpha(y)$ represents a generalised weighting given by

$$\rho_\alpha(y) = \frac{P(y)^\alpha}{\sum_y P(y)^\alpha}. \quad [2]$$

For $\alpha \neq 1$, f can be any invertible function positive in $[0, \infty)$ which is a linear transform of

$$f(x) = 2^{(1-\alpha)x}. \quad [3]$$

Using this definition of conditional entropy ensures that entropy is additive (1, 2)

$$H_\alpha[X, Y] = H_\alpha[X] + H_\alpha[Y|X], \quad [4]$$

where $H_\alpha[X, Y]$ represents the joint Renyi entropy of two random variables defined as

$$H_\alpha[X, Y] = \frac{1}{1-\alpha}\log\left(\sum_{x,y} P(x, y)^\alpha\right). \quad [5]$$

For simplicity, we will use the following definition for the conditional Renyi entropy of order $\alpha = 2$

$$H_2[X|Y] = -\log\left(\sum_y \rho_2(y) 2^{-H_2[X|y]}\right). \quad [6]$$

Comparing this expression to the probability of coincidence suggests the following definition for the conditional probability of coincidence averaged over Y

$$p_C[X|Y] = \sum_y \rho_2(y) p_C[X|y], \quad [7]$$

such that

$$H_2[X|Y] = -\log p_C[X|Y]. \quad [8]$$

This definition appears quite natural as it ensures that the conditional probability of coincidence behaves like a regular conditional probability

$$p_C[X|Y] = \frac{p_C[X, Y]}{p_C[Y]}, \quad [9]$$

and follows Bayes' theorem

$$p_C[X|Y] = \frac{p_C[Y|X] p_C[X]}{p_C[Y]}. \quad [10]$$

2. Relations between interaction information and conditional mutual information

Interaction information and conditional mutual information can be related using the additive property of entropy to give

$$I_{2,int}(X, Y|\Pi) = I_2(X, \Pi|Y) - I_2(X, \Pi) \quad [11]$$
$$= I_2(Y, \Pi|X) - I_2(Y, \Pi). \quad [12]$$

Therefore, if $I_{2,int}(X, Y|\Pi) > 0$ then X and Y are more relevant when considered in the context of one another than when taken independently. Conversely, for $I_{2,int}(X, Y|\Pi) < 0$ features are partially redundant and becomes less informative in the context of one another. Interaction information may also be expressed in terms of the coincidence mutual information between X and Y to give

$$I_{2,int}(X, Y|\Pi) = I_2(X, Y|\Pi) - I_2(X, Y) \quad [13]$$
$$= I_2(Y, X|\Pi) - I_2(Y, X). \quad [14]$$

Features with positive interaction information are those which become more informative about one another in the context of Π. Features with zero interaction information experience no change in their mutual information, while features with negative

interaction information have a decrease in their mutual information. Finally, conditional mutual information may be expressed as

$$I_2(X,\Pi|Y) = I_2(X,\Pi) + I_{2,int}(X,Y|\Pi). \quad [15]$$

So that the information X provides about Π in the context of Y is equal to the information it provides on its own plus a coupling term determined by its interactions with Y. If one feature makes another fully redundant, e.g. $I_2(X,\Pi|Y) = 0$, then the interaction information between them is $I_{2,int}(X,Y|\Pi) = -I_2(X,\Pi)$ such that their coupling completely negates the information provided by X on its own. As explored in (3), definitions of redundancy and synergy may be built from interaction information and conditional mutual information. Interaction information captures a mixture of synergy and redundancy and is positive if synergy outweighs redundancy, negative if redundancy outweighs synergy and zero if they are both equal.

3. Linking pairwise classification odds to coincidence information

A. Likelihood ratio in terms of coincidence probabilities. We begin with the posterior odds

$$\frac{P(\pi|x=x')}{P(B|x=x')} = \frac{P(x=x'|\pi)}{P(x=x'|B)}\frac{P(\pi)}{P(B)}. \quad [16]$$

Similarly to (4) we may relate the probability distributions associated with π and B using the following expression: $P(x|\pi) = Q^\pi(x)P(x|B)$, where $Q^\pi(x)$ is a selection factor which reweighs the probability of each feature outcome according to some fitness function. To ensure normalisation of $P(X|\pi)$, we require that $\langle Q^\pi(x)\rangle_{P(x|B)} = 1$. $\langle\rangle_{P(x|B)}$ indicates an expectation value over the distribution $P(X|B)$. $P(x=x'|\pi)$ is the probability of a match in feature X if both sequences were truly drawn from distribution $P(\Sigma|\pi)$. We compute this by summing over all possible ways to obtain such a match

$$P(x=x'|\pi) = \sum_{x,x'} \delta_{x,x'} P(x|\pi)P(x'|\pi) = p_C[X|\pi], \quad [17]$$

where $\delta_{x,x'}$ is the Kronecker delta. $P(x=x'|B)$ is the probability of a match in feature X for a query drawn from $P(\Sigma|B)$ and a reference drawn from $P(\Sigma|\pi)$. This may be written as

$$P(x=x'|B) = \sum_{x,x'} \delta_{x,x'} P(x|\pi)P(x'|B). \quad [18]$$

Using the fact that $P(x|\pi) = Q^\pi(x)P(x|B)$ this may be rewritten as

$$P(x=x'|B) = \sum_{x,x'} \delta_{x,x'} Q^\pi(x)P(x|B)P(x'|B), \quad [19]$$

which may also be written as

$$\begin{aligned} P(x=x'|B) &= \left\langle \sum_{x'} \delta_{x,x'} Q^\pi(x)P(x'|B) \right\rangle_{P(x|B)} \\ &= \langle Q^\pi(x)P(x|B)\rangle_{P(x|B)}. \end{aligned} \quad [20]$$

Assuming that the background probability of a particular feature is independent of its selection factor, we may decompose the expectation value to arrive at

$$\begin{aligned} P(x=x'|B) &= \langle Q^\pi(x)\rangle_{P(x|B)} \langle P(x|B)\rangle_{P(x|B)} \\ &= p_C[X|B] \end{aligned} \quad [21]$$

As $\langle Q^\pi(x)\rangle_{P(x|B)} = 1$ by normalisation. We will write $p_C[X|B] = p_C[X]$ as it is the probability of coincidence over a background distribution.

B. Average classification odds over subsets of specific TCRs. The odds ratio for a particular epitope subset given a match in feature X is:

$$\frac{P(\pi|x=x')}{P(B|x=x')} = \frac{p_C[X|\pi]}{p_C[X]}\frac{P(\pi)}{P(B)} \quad [22]$$

Taking the average of this over the normalised distribution of epitopes $P(\Pi)/(1-P(B))$ yields

$$\sum_\pi \frac{P(\pi|x=x')}{P(B|x=x')}\frac{P(\pi)}{1-P(B)} = \sum_\pi \frac{p_C[X|\pi]}{p_C[X]}\frac{P(\pi)}{P(B)}\frac{P(\pi)}{1-P(B)}. \quad [23]$$

Recalling the definition of the conditional probability of coincidence, Eq. 6, the right hand side becomes

$$\sum_{\pi} \frac{p_C[X|\pi]}{p_C[X]} \frac{P(\pi)}{P(B)} \frac{P(\pi)}{1-P(B)} = \frac{p_C[X|\Pi]}{p_C[X]} \frac{\sum_{\pi'} P(\pi')^2}{P(B)(1-P(B))}, \quad [24]$$

we therefore obtain in full:

$$\sum_{\pi} \frac{P(\pi|x=x')}{P(B|x=x')} \frac{P(\pi)}{1-P(B)} = \frac{p_C[X|\Pi]}{p_C[X]} \frac{\sum_{\pi'} P(\pi')^2}{P(B)(1-P(B))}, \quad [25]$$

which may be written as:

$$\left\langle \frac{P(\pi|x=x')}{P(B|x=x')} \right\rangle = \frac{p_C[X|\Pi]}{p_C[X]} \left\langle \frac{P(\pi)}{P(B)} \right\rangle. \quad [26]$$

Where $\langle\rangle$ denotes an average over $\frac{P(\pi)}{1-P(B)}$.

C. Relating the probability of specificity and the probability of being drawn from a specific subset. We show here how $P(\pi)$ and $P(\pi|x=x')$, which represent the prior and posterior probabilities that a sequence is observed because it was drawn from distribution $P(\Sigma|\pi)$ may be related to the prior and posterior probabilities that a sequence is in-fact specific to epitope π. We consider the mixture distribution of sequences

$$P(\sigma) = P(\pi)P(\sigma|\pi) + P(B)P(\sigma|B). \quad [27]$$

We denote the fraction of sequences in this mixture which would bind to epitope π in a test of specificity as $P(S^\pi)$. We may express this fraction in terms of the mixture proportions

$$P(S^\pi) = P(\pi)P(S^\pi|\pi) + P(B)P(S^\pi|B), \quad [28]$$

where $P(S^\pi|\pi)$ and $P(S^\pi|B)$ are the fractions of sequences produced by distributions $P(\Sigma|\pi)$ and $P(\Sigma|B)$ which are specific to π respectively. As $P(\Sigma|\pi)$ represents the distribution of sequences specific to π, by definition $P(S^\pi|\pi) = 1$, so

$$P(S^\pi) = P(\pi) + P(B)P(S^\pi|B). \quad [29]$$

As $P(\pi) + P(B) = 1$, this may be written as

$$P(S^\pi) = P(\pi) + (1-P(\pi))P(S^\pi|B). \quad [30]$$

If we assume that the fraction of sequences generated by distribution $P(\Sigma|B)$ which are specific to π is much smaller than the proportion $P(\pi)$ in our mixture so $P(S^\pi|B) \ll P(\pi)$, then we may simplify this to

$$P(S^\pi) \approx P(\pi). \quad [31]$$

Now substituting $P(S^\pi)$ into our prior odds in Eq. 14, we obtain

$$\frac{P(\pi|x=x')}{P(B|x=x')} = \frac{p_C[X|\pi]}{p_C[X]} \frac{P(S^\pi)}{1-P(S^\pi)}. \quad [32]$$

Following similar reasoning, we may write the posterior probability that a sequence sampled from the mixture is specific to epitope π given that it matches in feature X with sequence with known specificity to π as

$$P(S^\pi|x=x') = P(\pi|x=x')P(S^\pi|\pi, x=x') + P(B|x=x')P(S^\pi|B, x=x'). \quad [33]$$

Once again, $P(S^\pi|\pi, x=x') = 1$ and $P(S^\pi|B, x=x') \leq 1$, therefore

$$P(S^\pi|x=x') \approx P(\pi|x=x') \quad [34]$$

as long as the posterior odds exceeds one. Taken together, this allows us to re-express Eq. 14 in terms of prior and posterior odds of specificity to π

$$\frac{P(S^\pi|x=x')}{1-P(S^\pi|x=x')} = \frac{p_C[X|\pi]}{p_C[X]} \frac{P(S^\pi)}{1-P(S^\pi)}. \quad [35]$$

D. Simulating pairwise classification with limited information.

D.1. Generating background and specific TCRs. Here, we followed a previously described procedure for the in silico simulation of epitope-specific repertoires (4). In short, we simulated a synthetic background repertoire by randomly generating strings of length k from a predefined alphabet of q characters. We defined 'epitope specific' TCRs by a set of hard coded rules: For each epitope we generated M motifs, where each motif contained c out of q letters for each of the TCR's k 'residues'. To generate the specific TCRs for a motif, we produced all possible combinations of these amino acids. The set of TCRs specific to a given epitope was the full set of TCRs produced by all motifs associated with the epitope.

D.2. Comparing theoretical perforance to true classifcation accuracy. We computed the relevancy of various 'features' of simulated sequences for $q = 20$, $k = 4$, $M = 5$ and $c = 3$. To define features we divided each TCR by its residues. For example, to produce an α and a β chain we divided each TCR into two halves. We then produced a mixture data set into which we mixed a particular fraction of simulated background and specific TCRs. For each TCR we also assigned a label identifying which of these two original sets it came from. We then simulated pairwise classification using feature matching by sampling sequences from the mixture set and looking for matches in a particular feature with a TCR from the true set of specific sequences. Using the labels, we were able to calculate the probability that the sampled sequence was truly specific given that this match occurred. We performed this simulation using a range of features and mixture ratios as shown in Figure S1 and simulation results aligned with the theoretical predictions from coincidence information.

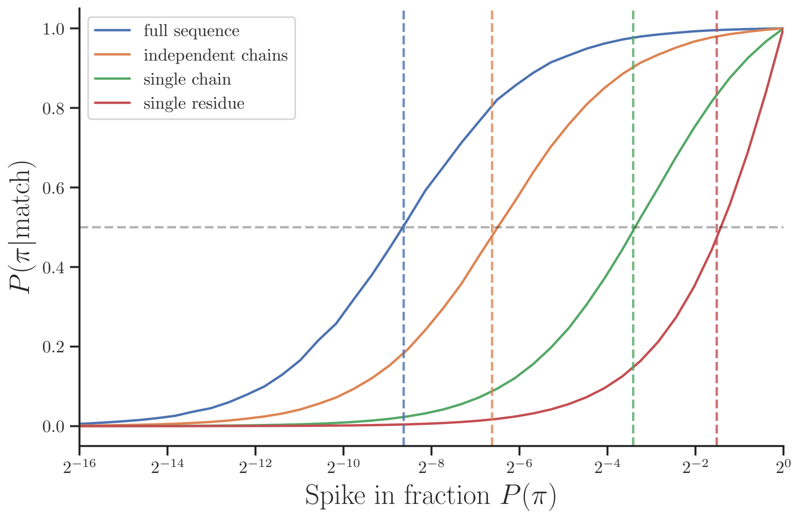

Fig. S1. Simulated prior vs posterior probabilities given feature match Prior probability of a sequence being present in a mixture distribution due to being sampled from a spiked in epitope specific distribution, vs posterior probability given a feature match with a specific sequence. Dashed lines indicate theoretical prior probability values for which a posterior probability of 0.5 should be observed given the informational value of a given feature computed from Eq. 17. Solid curves cover classification for a given fraction of spiked in specific sequence in the following scenarios: using matches in the full simulated TCR (single chain), using matches in one half of the TCR (single chain), using matches from a single amino acid (single residue), using matches in both halves of the TCR but considered independently (independent chains).

E. Classification odds given a fuzzy match. For both query and reference being drawn from $P(\Sigma|\pi)$, the probability of a match is

$$P(d(x,x') = \Delta|\pi) = \sum_{x,x'} \delta_{d(x,x'),\Delta} P(x|\pi)P(x'|\pi) = p_C[X|\pi](\Delta). \quad [36]$$

Now consider the case where the query is truly from $P(\Sigma|B)$ while the reference is from $P(\Sigma|\pi)$

$$P(d(x,x') = \Delta|B) = \sum_{x,x'} \delta_{d(x,x'),\Delta}\ P(x|B)P(x'|\pi). \quad [37]$$

Similarly to the case for exact matching, we may write $P(x'|\pi) = Q^\pi(x')P(x'|B)$ such that

$$P(d(x,x') = \Delta|B) = \sum_{x,x'} \delta_{d(x,x'),\Delta}\ P(x|B)Q^\pi(x')P(x'|B). \quad [38]$$

Next, we may rewrite this equation as:

$$P(d(x,x') = \Delta|B) = \left\langle \sum_{x} \delta_{d(x,x'),\Delta}\ P(x|B)Q^\pi(x') \right\rangle_{P(x'|B)} \quad [39]$$

$$= \left\langle Q^\pi(x') \sum_{x} \delta_{d(x,x'),\Delta}\ P(x|B) \right\rangle_{P(x'|B)} \quad [40]$$

We define $n_\Delta(x') = \sum_x \delta_{d(x,x'),\Delta} P(x|B)$ to be the neighbourhood density around x' at distance Δ. Assuming independence of neighbor density $n_\Delta(x')$ and selection factor $Q^\pi(x')$ we have

$$P(d(x,x') = \Delta|B) = \left\langle Q^\pi(x') \right\rangle_{P(x'|B)} \left\langle n_\Delta(x') \right\rangle_{P(x'|B)}. \quad [41]$$

By normalization the first term evaluates to 1 so we are left with

$$P(d(x,x')=\Delta|B) = \left\langle n_\Delta(x')\right\rangle_{P(x'|B)} = \sum_{x,x'} \delta_{d(x,x'),\Delta} P(x|B)P(x'|B) = p_C[X|B](\Delta), \quad [42]$$

therefore the likelihood ratio becomes

$$\frac{P(d(x,x')=\Delta|\pi)}{P(d(x,x')=\Delta|B)} = \frac{p_C[X|\pi](\Delta)}{p_C[X](\Delta)}. \quad [43]$$

Where once again we have used $p_C[X](\Delta) = p_C[X|B](\Delta)$ to denote the background probability of coincidence.

4. Higher order Renyi entropies

A. Relationship to Hill numbers and generalised coincidence probabilities. From the definition of Renyi entropies in Eq. 3 it follows that Renyi entropies of order α are linked to Hill numbers in ecology, and generalised coincidence probabilities

The Renyi entropy of order α may be written as

$$H_\alpha[X] = \log D_\alpha[X], \quad [44]$$

where Hill numbers of order α are defined as (5)

$$D_\alpha[X] = \left(\sum_x P(x)^\alpha\right)^{\frac{1}{1-\alpha}}. \quad [45]$$

This generalises the relationship between H_2 and Simpson's diversity D_2. Hill numbers represent measures of the effective number of species in the distribution with increasing α giving higher weight to more probable outcomes. In ecological terms, each Hill number represents the reciprocal of a different mean of species proportional abundances. $\alpha = 1$ represents a geometric mean and $\alpha = 2$ an arithmetic mean.

Eq. 4 in the main text can also be generalised in terms of higher-order coincidence probabilities

$$H_\alpha[X] = \frac{1}{1-\alpha} \log p_\alpha[X], \quad [46]$$

where

$$p_\alpha[X] = \sum_x P(x)^\alpha, \quad [47]$$

is the probability that α independent draws from $P(X)$ return the same outcome, such that $p_C[X] = p_2[X]$.

B. Generalising the classification odds interpretation. In analogy to Eq. 5 we define

$$p_\alpha[X|Y] = \sum_y \rho_\alpha(x) p_\alpha[X|y], \quad [48]$$

so that from the proposed definition of conditional Renyi entropy (SI Text 1) it follows that

$$\begin{aligned} H_\alpha[X|Y] &= \frac{1}{1-\alpha} \log\left(\sum_y \rho_\alpha(y) 2^{(1-\alpha)H_\alpha[X|y]}\right) \\ &= \frac{1}{1-\alpha} \log p_\alpha[X|Y]. \end{aligned} \quad [49]$$

We now consider a classification procedure in which we have N query sequences and a single reference sequence drawn from $P(\Sigma|\pi)$ which all match in feature X. The posterior odds that all queries were drawn from $P(\Sigma|\pi)$ as opposed to $P(\Sigma|B)$ given this match may be written as

$$\frac{P(\pi|x=x'=...=x^{(N)})}{P(B|x=x'=...=x^{(N)})} = \frac{P(x=x'=...=x^{(N)}|\pi)}{P(x=x'=...=x^{(N)}|B)} \left(\frac{P(\pi)}{P(B)}\right)^N. \quad [50]$$

As in the case of a single query, the probability that all queries and the reference match given that they were all drawn from $P(\Sigma|\pi)$ is

$$P(x=x'=...=x^{(N)}|\pi) = \sum_{x,x',...,x^{(N)}} \delta_{x,x',...,x^{(N)}} P(x|\pi)P(x'|\pi)...P(x^{(N)}|\pi) = \sum_x P(x|\pi)^{N+1}, \quad [51]$$

while the probability that they all match given that the reference was drawn from $P(\Sigma|\pi)$ but all queries were drawn from $P(\Sigma|B)$ is

$$P(x=x'=...=x^{(N)}|B) = \sum_{x,x',...,x^{(N)}} \delta_{x,x',...,x^{(N)}} P(x|\pi)P(x'|B)...P(x^{(N)}|B) = \sum_{x,x'} \delta_{x,x'} P(x|\pi)P(x'|B)^N. \quad [52]$$

As before, writing $P(x|\pi)$ in terms of the background probability and a selection factor we get

$$P(x = x' = ... = x^{(N)}|B) = \sum_{x,x'} \delta_{x,x'} Q^{\pi}(x) P(x|B) P(x'|B)^N, \quad [53]$$

which may be written as

$$\begin{aligned} P(x = x' = ... = x^{(N)}|B) &= \left\langle \sum_{x'} \delta_{x,x'} Q^{\pi}(x) P(x'|B)^N \right\rangle_{P(x|B)} \\ &= \left\langle Q^{\pi}(x) P(x|B)^N \right\rangle_{P(x|B)}. \end{aligned} \quad [54]$$

We once again assume total independence of background probability and selection factor

$$\begin{aligned} P(x = x' = ... = x^{(N)}|B) &= \langle Q^{\pi}(x)\rangle_{P(x|B)} \left\langle P(x|B)^N \right\rangle_{P(x|B)} \\ &= \sum_{x} P(x|B)^{N+1}. \end{aligned} \quad [55]$$

Therefore the classification odds may be written as

$$\frac{P(\pi|x = x' = ... = x^{(N)})}{P(B|x = x' = ... = x^{(N)})} = \frac{p_{N+1}[X|\pi]}{p_{N+1}[X]} \left(\frac{P(\pi)}{P(B)}\right)^N, \quad [56]$$

where we have used Eq. 47. We now average over a distribution of epitope specific sets

$$\sum_{\pi} \frac{P(\pi|x = x' = ... = x^{(N)})}{P(B|x = x' = ... = x^{(N)})} \frac{P(\pi)}{1 - P(B)} = \sum_{\pi} \frac{p_{N+1}[X|\pi]}{p_{N+1}[X|B]} \left(\frac{P(\pi)}{P(B)}\right)^N \frac{P(\pi)}{1 - P(B)}. \quad [57]$$

Using Eq. 48 the right hand side becomes

$$\sum_{\pi} \frac{p_{N+1}[X|\pi]}{p_{N+1}[X]} \left(\frac{P(\pi)}{P(B)}\right)^N \frac{P(\pi)}{1 - P(B)} = \frac{p_{N+1}[X|\Pi]}{p_{N+1}[X]} \frac{\sum_{\pi'} P(\pi')^{N+1}}{P(B)^N (1 - P(B))}. \quad [58]$$

Therefore we obtain

$$\left\langle \frac{P(\pi|x = x' = ... = x^{(N)})}{P(B|x = x' = ... = x^{(N)})} \right\rangle = \frac{p_{N+1}[X|\Pi]}{p_{N+1}[X]} \left\langle \left(\frac{P(\pi)}{P(B)}\right)^N \right\rangle. \quad [59]$$

Using Eq. 46 and Eq. 49 we may rewrite this as

$$\begin{aligned} \left\langle \frac{P(\pi|x = x' = ... = x^{(N)})}{P(B|x = x' = ... = x^{(N)})} \right\rangle &= \left(\frac{2^{H_{N+1}[X]}}{2^{H_{N+1}[X|\Pi]}}\right)^N \left\langle \left(\frac{P(\pi)}{P(B)}\right)^N \right\rangle \\ &= 2^{N\left(H_{N+1}[X] - H_{N+1}[X|\Pi]\right)} \left\langle \left(\frac{P(\pi)}{P(B)}\right)^N \right\rangle \end{aligned} \quad [60]$$

Therefore, the change in entropy of a single bit between $H_{N+1}[X]$ and $H_{N+1}[X|\Pi]$ leads to an average 2^N fold increase in posterior odds if classifying using a match between N queries and a single reference.

5. The relevance of CDR3 length and net charge increases when conditioning

A striking feature of many epitope specific repertoires is the co-existence of several clusters with additional more dispersed TCRs (4, 6). The existence of multiple binding solutions, with presumably partially differing requirements on the CDR3 features required for specific binding, can 'wash out' the relevance of otherwise informative features. Here, we show how the comparison of total and conditional mutual information can help pinpoint instances of this phenomenon at work.

We anticipated that CDR3α and CDR3β sequence lengths should be relatively informative features as they directly impact the number of possible TCR-epitope contacts. We instead identified that length relevancies were relatively low (Fig. S3). We hypothesised that if we were to condition on a feature which sets the structural framework a CDR3 would have to operate in, then we would see an increased CDR3 length relevancy. We posited that V and J gene usage could provide suitable conditioning variables, as the gene usage determines framework and CDR1/2 variability. Our analyses confirmed this hypothesis and we found that CDR3 lengths provided substantially more information when conditioning on VJ gene usage (Fig. S2). We also found a similar increase in CDR3 net charge relevancies when conditioning on VJ usage. This shows that in epitope specific TCRs CDR3 length and net charge is highly restricted within VJ-matched TCRs in a manner not present in background sequences, and only more subtly when considering all specific TCRs.

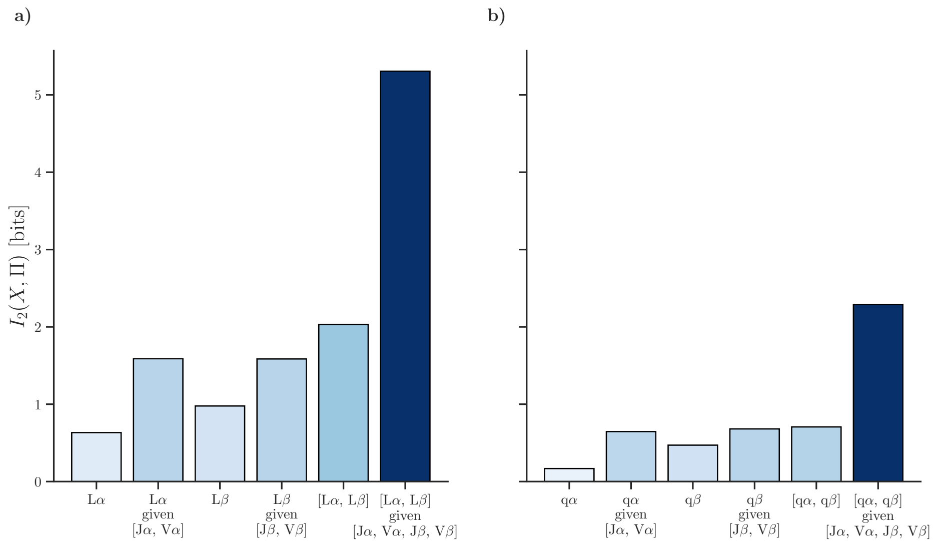

Fig. S2. Information of CDR3 length and net charge conditional on VJ usage. a) Relevancy of CDR3 length when considered independently and in the context of the V and J genes. While both CDR3α and CDR3β length are slightly informative features and show some synergy, they increase in relevancy significantly when considered in the context of the V and J genes. **b)** Relevancy of CDR3 net charge when considered independently and in the context of the V and J genes. As with length, CDR3 net charge increases in relevancy significantly in the context of the V and J genes.

6. Feature interactions arising from data structure

A. Mixture of motifs model. In the following we explore in a minimal model how local feature relevancy and interaction information depend on the number of distinct binding solutions able to engage a particular epitope. We consider that for a given epitope π there is a distribution of motifs/binding modes, $P(\Phi|\pi)$. For a given binding mode, $\Phi = \phi$, there is an associated set of specific sequences and sequence features, $P(X|\phi, \pi)$. The probability of drawing a particular feature $X = x$ from the distribution of sequences specific to π is

$$P(x|\pi) = \sum_{\phi} P(x|\phi, \pi)P(\phi), \qquad [61]$$

The coincidence probability for sequences drawn from $P(X|\pi)$ is

$$p_C[X|\pi] = \sum_{x} \left(\sum_{\phi} P(x|\phi, \pi)P(\phi) \right)^2 \qquad [62]$$

In (4) we showed that such a mixture distribution may be expressed as the sum of a within-motif and a cross-motif term

$$p_C[X|\pi] = p_C[\Phi] \left\langle p_C[X|\pi, \phi] \right\rangle_{P(\phi|\phi_1=\phi_2=\phi)} + (1 - p_C[\Phi]) \left\langle p_C[X|\pi, \phi_1; X|\pi, \phi_2] \right\rangle_{P(\phi_1,\phi_2|\phi_1\neq\phi_2)}, \qquad [63]$$

where $\langle p_C[X|\pi, \phi] \rangle_{P(\phi|\phi_1=\phi_2=\phi)} = p_C[X|\pi, \Phi]$ is the average probability of feature coincidence within each motif subset for motifs associated with epitope π weighted by

$$P(\phi|\phi_1 = \phi_2 = \phi) = \frac{P(\phi)^2}{\sum_{\phi'} P(\phi')^2} = \rho_2(\phi). \qquad [64]$$

And $\langle p_C[X|\pi, \phi_1; X|\pi, \phi_2] \rangle_{P(\phi_1,\phi_2|\phi_1\neq\phi_2)}$ is the cross-motif coincidence probability, where the probability of coincidence across two distributions $P(X)$, $P(Y)$ is defined as:

$$p_C[X; Y] = \sum_{x,y} P(x)P(y)\delta_{x,y}, \qquad [65]$$

and where the average is computed with respect to the following weighting factor

$$P(\phi_1, \phi_2|\phi_1 \neq \phi_2) = \frac{P(\phi_1)P(\phi_2)}{1 - \sum_{\phi'} P(\phi')^2}. \qquad [66]$$

We assume that in general this cross-motif coincidence probability is far smaller than the within coincidence probability such that

$$p_C[X|\pi] \approx p_C[\Phi] p_C[X|\pi, \Phi]. \qquad [67]$$

To proceed, we will now assume that $p_C[\Phi] = 1/M$, where M is number of motifs associated with a given epitope π. We will also assume that for a given M there is a characteristic probability of coincidence $p_C[X|\Pi_M]$ where Π_M denotes the fact that we are considering epitopes with M motifs. We use uppercase Π to highlight that we are theorising that epitopes with equal number of motifs only vary in their coincidence probabilities for particular features due to finite sample size error and hence their true coincidence probabilities should be equal in the limit of infinite data. We will assume that $p_C[X|\pi,\Phi] = p_C[X|\Pi_1]$ such that it is the probability of feature coincidence for epitopes with a single motif. Therefore, for an epitope with M motifs we obtain

$$p_C[X|\Pi_M] \approx \frac{1}{M} p_C[X|\Pi_1]. \qquad [68]$$

The local relevancy of X for an epitope with M motifs is then

$$\begin{aligned} i_2(X,\Pi_M) &= \log\left(\frac{1}{M}\frac{p_C[X|\Pi_1]}{p_C[X]}\right) \\ &= \log\left(\frac{p_C[X|\Pi_1]}{p_C[X]}\right) - \log M \\ &= i_2(X,\Pi_1) - \log M, \end{aligned} \qquad [69]$$

where $i_2(X,\Pi_1)$ is the characteristic local relevancy of feature X for epitopes with a single motif. Therefore, the greater the number of motifs associated with a given epitope, the less locally relevant each feature is expected to become. With such a model we would expect the local relevancy of two features, X and Y, for a given number of motifs to be related by the following equation

$$i_2(X,\Pi_1) - i_2(X,\Pi_M) = i_2(Y,\Pi_1) - i_2(Y,\Pi_M). \qquad [70]$$

Such that

$$i_2(X,\Pi_M) = i_2(Y,\Pi_M) + i_2(X,\Pi_1) - i_2(Y,\Pi_1). \qquad [71]$$

Which is a relationship we observe in Figure 5. Now let us consider how we expect the local interaction information between two features to change with number of motifs

$$\begin{aligned} i_{2,int}(X,Y|\Pi_M) &= i_2([X,Y],\Pi_M) - i_2(X,\Pi_M) - i_2(Y,\Pi_M) && [72] \\ &= i_2([X,Y],\Pi_1) - i_2(X,\Pi_1) - i_2(Y,\Pi_1) - \log M + 2\log M && [73] \\ &= i_{2,int}(X,Y|\Pi_1) + \log M. && [74] \end{aligned}$$

We expect the local interaction information between pairs of features to increase with the number of motifs associated to the particular eptiope. Therefore, we expect the interaction information between two features and their independent local relevancy for a given number of motifs to be related by the following expressions

$$\begin{aligned} i_{2,int}(X,Y|\Pi_M) - i_{2,int}(X,Y|\Pi_1) &= i_2(X,\Pi_1) - i_2(X,\Pi_M) && [75] \\ &= i_2(Y,\Pi_1) - i_2(Y,\Pi_M). && [76] \end{aligned}$$

So that

$$\begin{aligned} i_{2,int}(X,Y|\Pi_M) &= -i_2(X,\Pi_M) + i_2(X,\Pi_1) + i_{2,int}(X,Y|\Pi_1) && [77] \\ &= -i_2(Y,\Pi_M) + i_2(Y,\Pi_1) + i_{2,int}(X,Y|\Pi_1). && [78] \end{aligned}$$

Which is a relationship observed in Figure 5.

B. False positives. We will briefly show that false positives, that is sequences which are not truly specific to a given epitope but yet appear in a specific TCR sample, lead to similar results to the mixture of motif models. Consider that a sample of sequences is taken in an experiment designed to obtain sequences specific to epitope π and that a distribution of a particular feature X is obtained, $P(X|\tilde{\pi})$. A fraction of features in this distribution are truly specific to π, $P(\pi)$, while a fraction of sequences are false positives, $P(\neg\pi)$. We will denote the subset of sequence features truly specific to epitope π as $P(X|\pi)$ and the subset of sequence feature not specific to π as $P(X|\neg\pi)$. The probability of coincidence for the distribution $P(X|\tilde{\pi})$ is

$$p_C[X|\tilde{\pi}] = \sum_x \left(P(x|\pi)P(\pi) + P(x|\neg\pi)P(\neg\pi)\right)^2. \qquad [79]$$

Expanding out we get

$$\begin{aligned} p_C[X|\tilde{\pi}] &= \sum_x P(X|\pi)^2 P(\pi)^2 + \sum_x P(X|\neg\pi)^2 P(\neg\pi)^2 + 2\sum_x P(X|\pi)P(X|\neg\pi)P(\pi)P(\neg\pi) && [80] \\ &= P(\pi)^2\, p_C[X|\pi] + P(\neg\pi)^2\, p_C[X|\neg\pi] + 2P(\pi)P(\neg\pi)\, p_C[X|\pi; X|\neg\pi]. && [81] \end{aligned}$$

We will assume that cross-coincidences are rare, that coincidences are far more likely between the truly specific sequences and that the fraction of false positives is far smaller than the fraction of true positives such that the following expressions hold: $P(\pi)^2 p_C[X|\pi] \gg 2P(\pi)P(\neg\pi)p_C[X|\pi; X|\neg\pi]$ and $P(\pi)p_C[X|\pi] \gg P(\neg\pi)p_C[X|\neg\pi]$. Therefore we obtain

$$p_C[X|\tilde{\pi}] \approx P(\pi)^2 p_C[X|\pi].$$

Now considering the local relevancy of X computed from $p_C[X|\tilde{\pi}]$

$$\begin{aligned} i_2(X, \tilde{\pi}) &= \log\left(\frac{P(\pi)^2 \, p_C[X|\pi]}{p_C[P(X)]}\right) \\ &= \left(\frac{p_C[X|\pi]}{p_C[X]}\right) + 2\log P(\pi) \\ &= i_2(X, \pi) + 2\log P(\pi). \end{aligned} \qquad [82]$$

If $P(\pi) < 1$, then $\log P(\pi) < 0$ so the relevancy of X will be lower when computed using distribution $P(X|\tilde{\pi})$ than when using $P(X|\pi)$. Similarly to A, we identify that when $P(\pi) < 1$ the local interaction information between two features X and Y will be increased

$$\begin{aligned} i_{2,int}(X, Y|\tilde{\pi}) &= i_2([X, Y], \tilde{\pi}) - i_2(X, \tilde{\pi}) - i_2(Y, \tilde{\pi}) \\ &= i_2([X, Y], \pi) - i_2(X, \pi) - i_2(Y, \pi) + 2\log P(\pi) - 2\log P(\pi) - 2\log P(\pi) \\ &= i_{2,int}(X, Y|\pi) - 2\log P(\pi). \end{aligned} \qquad [83]$$

Therefore the relationships observed in Figure 5 could also be produced by false positives. Analyses of how TCR clustering depends on background sequence space coverage could provide a potential avenue for distinguishing between both models in future work.

7. Data analysis methods

A. Data collection and pre-processing. TCR sequencing data from Minervina et al. (7) and Dash et al. (6) was processed as follows: Data was deduplicated on the full nucleotide sequence level for each epitope in order to enable assessment of clonal convergence independently of clonal expansions within individual donors (4). Only TCRs with full CDR3, V gene and J gene annotations for both the α and β chain were retained, and V and J gene names were standardized using tidytcells (8). Furthermore, epitope specific sets were only retained in cases where at least one full TCR coincidence was observed. This was the case for all three epitopes studied by Dash et al. (6). In the Minervina et al. (7) dataset this filtering retained nine out of nineteen epitopes, and seven of the ten most deeply sampled repertoires. Background TCR sequence data was produced using the OLGA generation model (9). We generated 1000000 α and β chain sequences and then randomly paired these to produce paired chain background data. The final number of sequences retained is detailed in Table S1.

Table S1. TCR sequence data used in this study. The first column shows the epitope ID used throughout figures.

Epitope ID	Data set	Epitope	HLA	Sequence count
	OLGA	N/A	N/A	1000000
1	Minervina	NQKLIANQF	HLA-B*15:01	148
2	Minervina	DTDFVNEFY	HLA-A*01:01	88
3	Minervina	LLYDANYFL	HLA-A*02:01	53
4	Minervina	PTDNYITTY	HLA-A*01:01	155
5	Minervina	YLQPRTFLL	HLA-A*02:01	288
6	Minervina	FTSDYYQLY	HLA-A*01:01	450
7	Minervina	ALSKGVHFV	HLA-A*02:01	197
8	Minervina	LTDEMIAQY	HLA-A*01:01	398
9	Minervina	TTDPSFLGRY	HLA-A*01:01	1909
10	Dash	NLVPMVATV	HLA-A*02:01	67
11	Dash	GLCTLVAML	HLA-A*02:01	92
12	Dash	GILGFVFTL	HLA-A*02:01	249

B. Statistical analysis. Coincidence probabilities and their variance were computed using unbiased estimators described in (10). Conditional probabilities of coincidence were averaged over epitopes using Eq. 6 with an equal weighting assigned to each epitope specific subset. Background entropies of the paired chain TCRs were computed as the sum of α and β chain entropies, exploiting the independent pairing of chains in the computationally constructed background. Mutual information scores and higher order statistics such as synergy were then computed from these entropies using Eqs. 9 and 11. Orthogonal distance regression was performed as implemented in Scipy (11).

Commonly used reduced amino acid alphabets were obtained from Biopython, including the physico-chemical PC5 alphabet (aliphatic, aromatic, charged, tiny, diverse) and a family of alphabets of different sizes obtained by clustering of the BLOSUM50 substitution matrix due to Murphy et al. (12). Additionally, we used the top-three scoring alphabets from (13), as provided by the Pepdata library. These alphabets included the GBMR alphabet, obtained via clustering based on preservation of folding information (14), and the (H)SDM alphabets, obtained via clustering of substitution matrices derived from structural alignment of (homologous) proteins (15).

Amino acid properties were obtained from the AAindex database (16). For simplicity, we focused our analyses on the curated set of 43 properties from this database previously described by Mei et al. (17).

Table S2. Software packages used in this study.

Name	Version	Link
Tidytcells (8)	2.00	https://pypi.org/project/tidytcells/
Pyrepseq (4)	1.5	https://pypi.org/project/pyrepseq/
OLGA (9)	1.2.4	https://github.com/statbiophys/OLGA
Scipy (11)	1.11.3	https://pypi.org/project/scipy/
Pepdata	1.0.7	https://pypi.org/project/pepdata/
AAindex	1.1.2	https://pypi.org/project/aaindex/
Biopython (18)	1.75	https://biopython.org/

C. Hierarchical clustering of amino acids by biophysical properties. For each biophysical property, a distance matrix was constructed containing the absolute pairwise differences in property values between pairs of amino acids. For a given maximal number of clusters, amino acids were clustered by property distance matrices using Ward's method for hierarchical clustering in Scipy (11). Amino acids were then remapped to a new alphabet using cluster annotations. Note that sometimes the algorithm returns an optimal number of clusters smaller than the allowed maximum, for instance for net charge for $N > 3$.

D. Greedy discovery of an optimal two letter alphabet. Our goal was to identify the remapping of amino acids into an alphabet of size 2 which maximised the joint remapped CDR3α and CDR3β relevancy. Remappings were produced using two groups (Group 1 and Group 2). Initially all amino acids were assigned to Group 2. Our algorithm then searched for the most informative single amino acid to move to Group 1. The algorithm then iteratively searched for the next most informative amino acid to add to those already part of Group 1. This process was repeated until no improvement could be made by moving an additional amino acid to Group 1. When applied to the TCR dataset, the algorithm placed the amino acids GSYAEKIW in group 1 in the order of addition, while all remaining amino acids were placed in group 2.

Supplementary Data

Table S3. Joint CDR3α and CDR3β relevancy scores for reduced amino acid alphabets obtained by clustering with respect to biophysical properties. Properties are ordered within each property type by average score across alphabet sizes.

Property	Information of N letter alphabets [bits]				Property type
	2	3	4	5	
Residue accessible surface area in tripeptide	14.0	16.4	22.1	24.0	steric
Van der Waals parameter R_0	14.1	18.0	21.4	21.5	steric
Distance between Cα and centroid of side chain	12.2	18.9	20.5	23.5	steric
Radius of gyration of side chain	12.2	18.9	20.5	23.4	steric
Average volume of buried residue	14.0	16.1	21.8	21.9	steric
Normalized Van der Waals volume	14.0	16.1	21.8	21.9	steric
Graph shape index	13.6	17.9	19.7	21.8	steric
Average accessible surface area	13.9	15.7	19.6	21.2	steric
Side chain torsion angle	7.8	17.3	22.0	23.2	steric
STERIMOL maximum width of the side chain	7.5	17.3	21.7	23.7	steric
Value of $\theta(i)$	12.8	14.6	20.2	21.4	steric
Refractivity	7.9	16.3	21.9	22.1	steric
STERIMOL length of the side chain	9.6	17.5	18.1	22.2	steric
Van der Waals parameter epsilon	5.1	14.5	14.6	20.5	steric
Side-chain angle	7.8	10.0	13.6	20.7	steric
STERIMOL minimum width of the side chain	7.2	13.4	13.7	13.7	steric
Number of full nonbonding orbitals	13.7	18.0	18.9	19.5	hydrophobic
Solvation free energy	11.2	18.8	19.5	20.3	hydrophobic
Retention coefficient in HPLC pH 2.1	10.4	18.8	19.0	20.1	hydrophobic
Melting point	13.3	16.6	17.9	20.0	hydrophobic
Retention coefficient in TFA	12.2	14.6	17.7	22.4	hydrophobic
Number of hydrogen-bond donors	13.8	15.7	18.0	18.0	hydrophobic
Partition coefficient in thin-layer chromatography	11.4	13.3	19.4	19.8	hydrophobic
Partition energy	8.1	16.1	19.8	19.9	hydrophobic
Hydration number	6.3	16.6	19.7	19.8	hydrophobic
Retention coefficient at pH 2	12.3	15.2	16.5	17.8	hydrophobic
Free energy of solution in water	4.6	14.6	16.4	17.9	hydrophobic
Retention coefficient in HPLC pH 7.4	7.9	10.9	10.9	17.0	hydrophobic
NMR chemical shift of α carbon	13.3	17.6	20.1	23.0	electronic
Electron-ion interaction potential values	13.8	18.9	19.8	20.5	electronic
Polarity	10.1	18.7	19.8	20.6	electronic
αCH chemical shifts	10.4	15.8	20.0	21.8	electronic
pKCOOH	7.4	17.6	20.3	21.2	electronic
Localized electrical effect	6.6	17.3	19.8	20.1	electronic
pKNH2	13.2	14.9	15.1	18.1	electronic
αNH chemical shifts	3.7	15.1	17.2	17.4	electronic
Charge transfer donor capability	12.1	12.1	12.1	12.1	electronic
Charge transfer capability	12.1	12.1	12.1	12.1	electronic
Amphiphilicity index	4.8	12.7	13.3	13.3	electronic
Isoelectric point	5.3	9.0	9.4	18.7	electronic
Net charge	5.0	9.0	9.0	9.0	electronic
Positive charge	5.9	5.9	5.9	5.9	electronic
Negative charge	5.0	5.0	5.0	5.0	electronic

Supplementary Figures

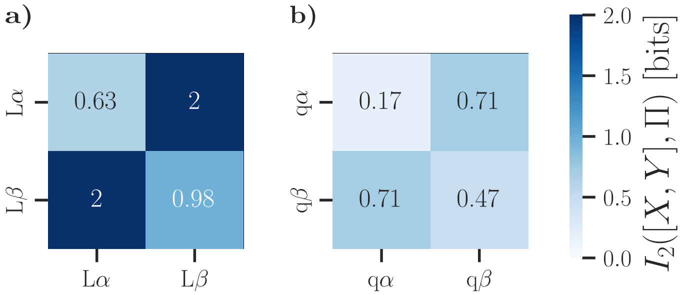

Fig. S3. Coincidence mutual information between CDR3 length/net charge and antigen specificity. Relevancy scores of CDR3α and CDR3β **a)** length and **b)** net charge. The off-diagonal values indicate the amount of coincidence information that combinations of features provide.

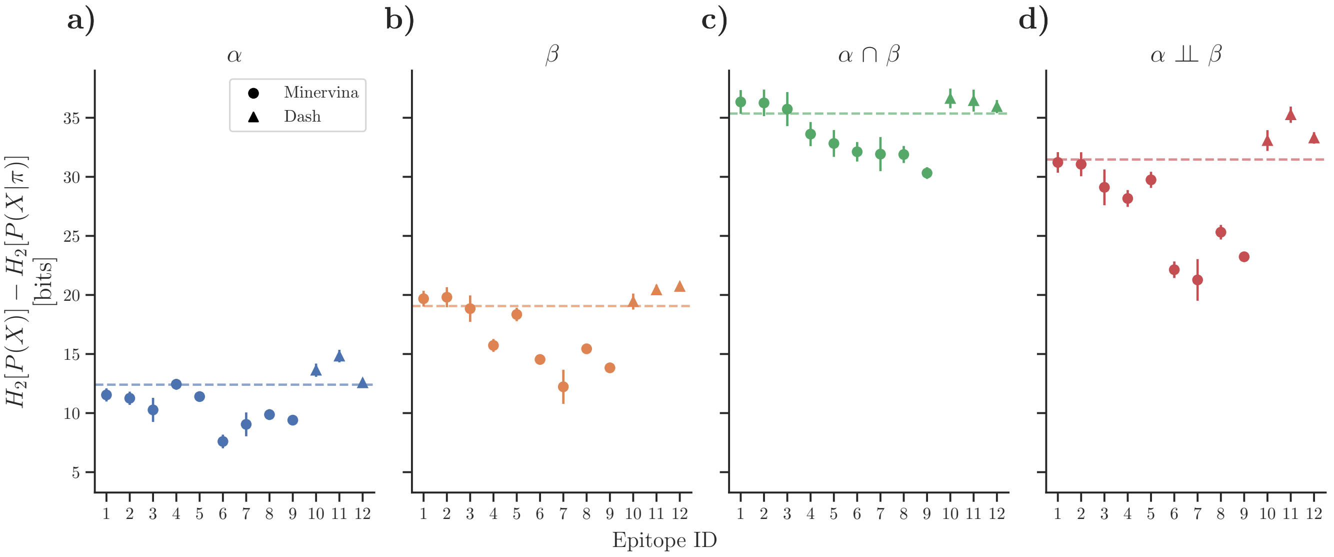

Fig. S4. Coincidence entropy reduction on the per-epitope level. Coincidence entropy reduction between specific and background TCRs for the **a)** α and **b)** β chains. **c)** Local relevancy of the full paired TCR chain. $X \cap Y$ represents joint consideration of features X and Y here and in the following. **d)** Sum of local relevancies of the α and β chain. $X \perp\!\!\!\perp Y$ represents separate consideration of features X and Y here and in the following. Epitopes are ordered within each dataset by their full entropy change. Dashed lines indicate the average entropy change shown in Figure 2. Although a large amount of variability between various epitopes not captured by their associated uncertainties is observed, every epitope shows a decrease in both α and β chain entropy. For every epitope, the α and β chain are therefore informative features. The change in entropy of the full TCR sequence $\alpha\beta$ is in all cases greater than the sum of the entropy changes of the α and β chains taken independently, suggesting that synergy between α and β chain information is a general phenomenon.

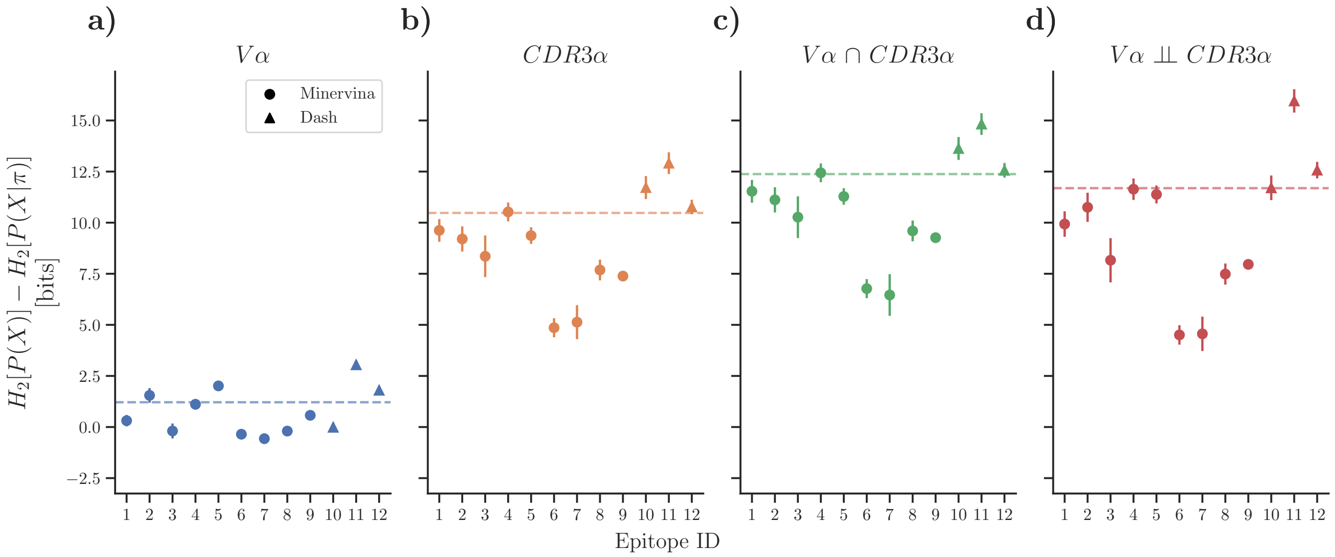

Fig. S5. Coincidence entropy reduction for CDR3α and Vα. Both the Vα and CDR3α contributions to specificity vary across epitopes, with some epitopes showing no detectable restriction of Vα gene choice. Further details as described for Fig. S4.

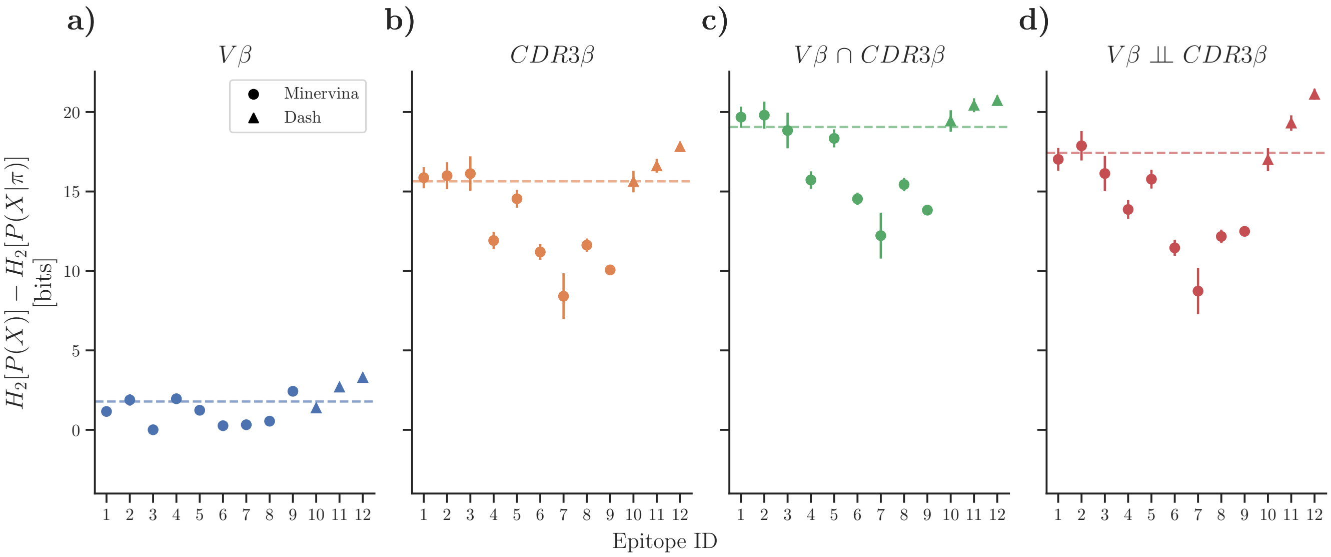

Fig. S6. Coincidence entropy reduction for CDR3β and Vβ. Both the Vβ and CDR3β contributions to specificity vary across epitopes. Most epitopes show small, but positive restriction of Vβ gene choice. Further details as described for Fig. S4.

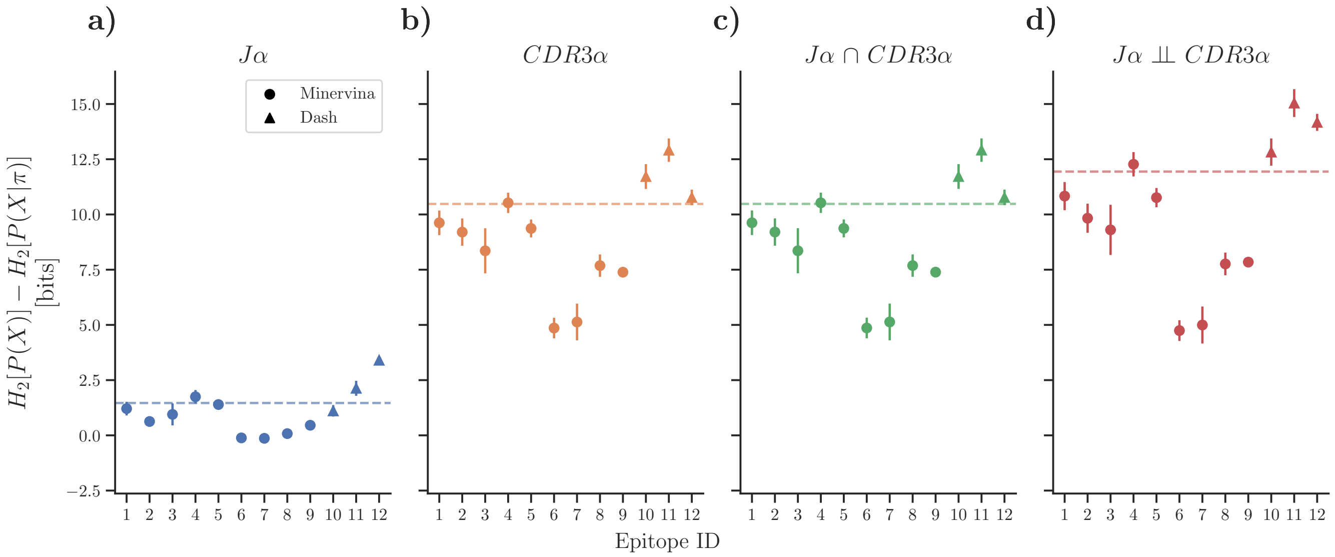

Fig. S7. Coincidence entropy reduction for CDR3α and Jα. Comparison of panel c and d reveals negative interaction information between Jα and CDR3α regions suggesting that the CDR3α makes the Jα redundant. Further details as described for Fig. S4.

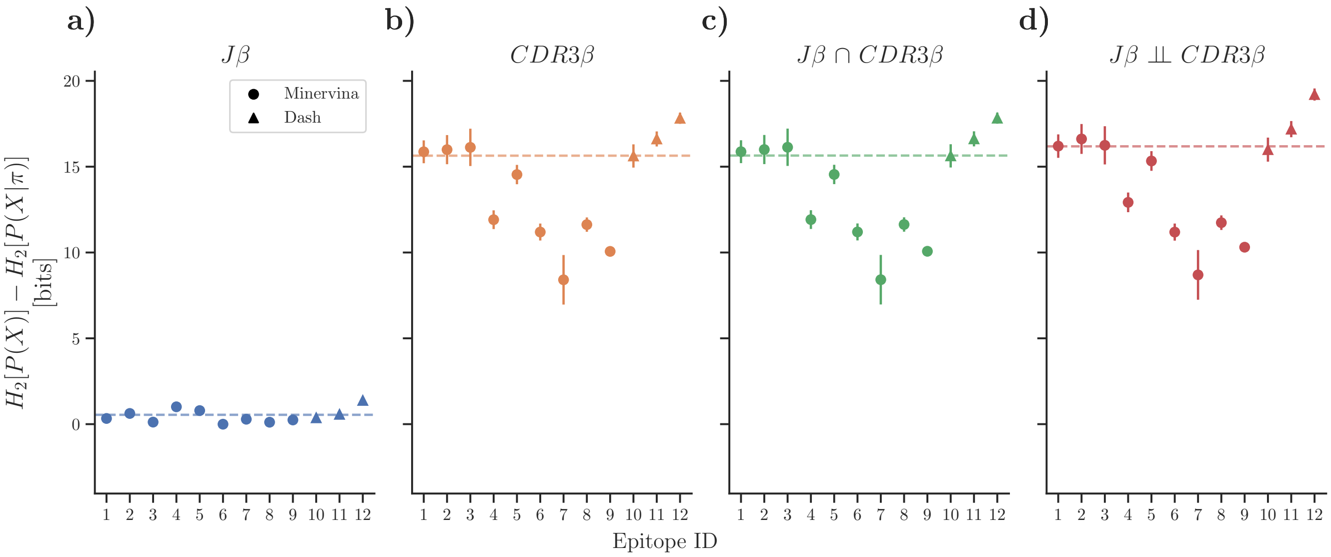

Fig. S8. Coincidence entropy reduction for CDR3β and Jβ. Comparison of panel c and d reveals negative interaction information between Jβ and CDR3β regions suggesting that the CDR3β makes the Jβ redundant. Further details as described for Fig. S4.

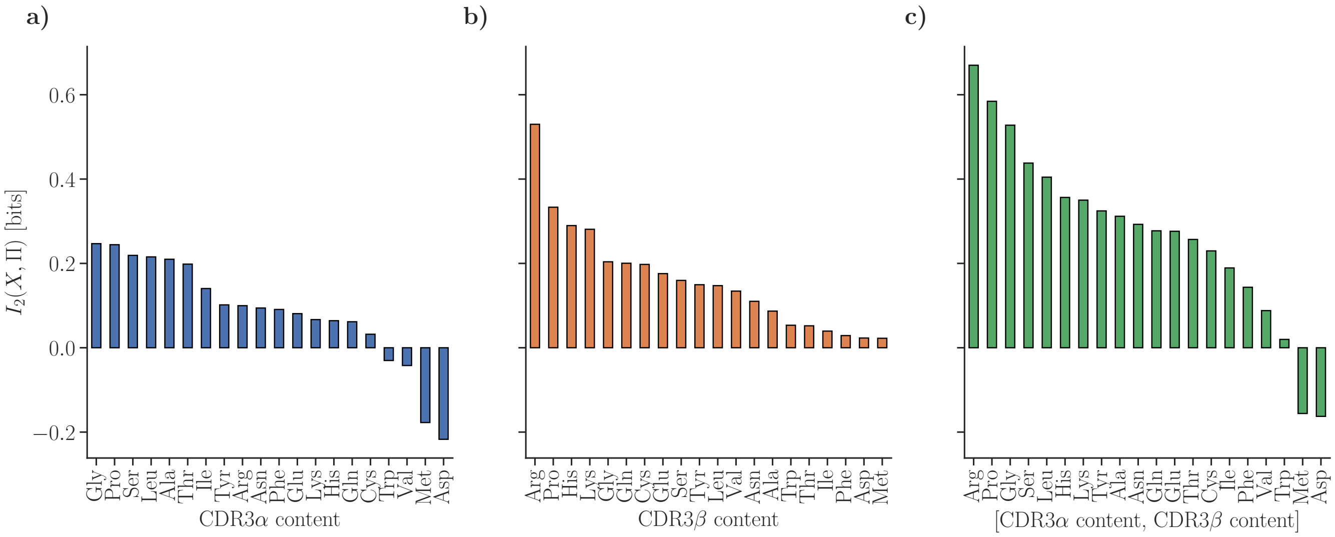

Fig. S9. Relevancy of amino acid contents. Information provided by knowing the number of times each particular amino acid occurs in a CDR3 sequence. Scores are shown for **a)** the α chain, **b)** the β chain and **c)** α and β chain contents considered jointly. Within each plot amino acids are ordered by their informativeness.

References

1. P Jizba, T Arimitsu, The world according to rényi: Thermodynamics of multifractal systems. *Annals Phys.* **312**, 17–59 (2004).
2. VM Ilić, MS Stanković, Generalized shannon–khinchin axioms and uniqueness theorem for pseudo-additive entropies. *Phys. A: Stat. Mech. its Appl.* **411**, 138–145 (2014).
3. PL Williams, RD Beer, Nonnegative decomposition of multivariate information. *arXiv preprint arXiv:1004.2515* (2010).
4. A Mayer, CG Callan Jr, Measures of Epitope Binding Degeneracy from T Cell Receptor Repertoires. *Proc. Natl. Acad. Sci.* **120**, e2213264120 (2023).
5. MO Hill, Diversity and evenness: A unifying notation and its consequences. *Ecology* **54**, 427–432 (1973).
6. P Dash, et al., Quantifiable predictive features define epitope-specific t cell receptor repertoires. *Nature* **547**, 89–93 (2017).
7. AA Minervina, et al., Sars-cov-2 antigen exposure history shapes phenotypes and specificity of memory cd8+ t cells. *Nat. Immunol.* **23**, 781–790 (2022).
8. Y Nagano, B Chain, tidytcells: standardizer for tr/mh nomenclature. *Front. Immunol.* **14**, 1276106 (2023).
9. Z Sethna, Y Elhanati, CG Callan Jr, AM Walczak, T Mora, Olga: Fast computation of generation probabilities of b-and t-cell receptor amino acid sequences and motifs. *Bioinformatics* **35**, 2974–2981 (2019).
10. A Tiffeau-Mayer, Unbiased estimation of sampling variance for simpson's diversity index. *Phys. Rev. E* **109**, 064411 (2024).
11. P Virtanen, et al., SciPy 1.0: Fundamental Algorithms for Scientific Computing in Python. *Nat. Methods* **17**, 261–272 (2020).
12. LR Murphy, A Wallqvist, RM Levy, Simplified amino acid alphabets for protein fold recognition and implications for folding. *Protein engineering* **13**, 149–152 (2000).
13. EL Peterson, J Kondev, JA Theriot, R Phillips, Reduced amino acid alphabets exhibit an improved sensitivity and selectivity in fold assignment. *Bioinformatics* **25**, 1356–1362 (2009).
14. AD Solis, S Rackovsky, Optimized representations and maximal information in proteins. *Proteins: Struct. Funct. Bioinforma.* **38**, 149–164 (2000).
15. A Prlić, FS Domingues, MJ Sippl, Structure-derived substitution matrices for alignment of distantly related sequences. *Protein Eng.* **13**, 545–550 (2000).
16. S Kawashima, et al., Aaindex: Amino acid index database, progress report 2008. *Nucleic acids research* **36**, D202–D205 (2007).
17. H Mei, ZH Liao, Y Zhou, SZ Li, A new set of amino acid descriptors and its application in peptide qsars. *Pept. Sci. Orig. Res. on Biomol.* **80**, 775–786 (2005).
18. PJ Cock, et al., Biopython: freely available python tools for computational molecular biology and bioinformatics. *Bioinformatics* **25**, 1422 (2009).
